# The chromatin architectural regulator SND1 mediates metastasis in triple-negative breast cancer by promoting CDH1 gene methylation

**DOI:** 10.1186/s13058-023-01731-3

**Published:** 2023-10-26

**Authors:** Huibian Zhang, Min Gao, Wenying Zhao, Lin Yu

**Affiliations:** 1 Department of Biochemistry and Molecular Biology, School of Basic Medical Sciences, Tianjin Medical University, Tianjin, 300070 China; 2 Laboratory of Molecular Immunology, Research Center of Basic Medical Science, Tianjin Medical University, Tianjin, 300070 China; 3 Tianjin Key Laboratory of Cellular and Molecular Immunology and Key Laboratory of the Educational Ministry of China, Tianjin Medical University, Tianjin, 300070 China

**Keywords:** SND1, Triple-negative breast cancer, Chromatin architectural interaction, Metastasis

## Abstract

**Background:**

SND1 participates in tumorigenesis, tumour invasion and metastasis in different cancers. Previous studies have shown that SND1 can promote the invasion and migration of breast cancer cells. Triple-negative breast cancer (TNBC) is a specific breast cancer subtype with high metastatic potential and poor prognosis. However, the specific roles and mechanisms of SND1 in TNBC metastasis remain unaddressed.

**Methods:**

Immunostaining was used to detect the SND1 expression in tissue samples of 58 TNBC and 10 glioblastomas (GBM) as positive control. The correlation between SND1 expression and patient prognosis was assessed using the Kaplan–Meier estimator. The gene expression was evaluated by qRT-PCR, Western blot and immunofluorescence analyses. Gene Ontology analysis, ChIP, a dual-luciferase reporter assay, EMSA, and 3C analysis were applied to identify SND1-activated target genes. Bisulfite sequencing PCR and MeDIP were used to detect DNA methylation. We also used wound healing, Transwell and orthotopic implantation assays to investigate the function of SND1 in TNBC cell migration and invasion.

**Results:**

The data of immunohistochemistry manifested that SND1 is the overexpression in metastasized TNBC and an independent factor for TNBC prognosis. SND1 knockdown inhibited the migration and invasion of TNBC cells. We found that SND1 promotes the metastatic phenotype of TNBC cells by epigenetically altering chromatin conformational interactions, which in turn activates DNMT3A transcription. Then, DNMT3A attenuates CCND1 expression by inducing CCND1 gene methylation, leading to TNBC metastasis.

**Conclusion:**

SND1 can promote the invasion and migration of TNBC cells by promoting DNMT3A expression and suppressing CDH1 activity. SND1 is a potential biomarker and a promising therapeutic target for TNBC.

**Supplementary Information:**

The online version contains supplementary material available at 10.1186/s13058-023-01731-3.

## Background

Breast cancer has the highest case morbidity and mortality in female cancer patients among the world [1, 2]. Triple-negative breast cancers (TNBC) is one of the most malignant and aggressive breast cancer [3, 4]. TNBC is highly invasive, and approximately 46% of TNBCs undergo distant metastasis, which is also the main factor for the mortality of patients [5, 6]. Due to the relationship between metastasis and TNBC prognosis, further investigation of the detail mechanism regulating TNBC metastasis is absolutely imperative.

Staphylococcal nuclease domain-containing 1 (SND1) is evolutionarily conserved and highly expressed in various cancers [7–9]. In different cancer types, SND1 promotes a malignant phenotype through different mechanisms. In colon and hepatocellular carcinoma, SND1 enhances the process capacity of RNA-induced silencing complex (RISC) [10, 11]. SND1 also promotes chemoresistance and epithelial-mesenchymal transition in ovarian cancer [9, 12]. In breast cancer, SND1 could interact with MTDH and promoter cancer progression [13, 14]. Our previous results indicated that SND1 can enhance histone acetylation in the promoters of TGFβ pathway-related genes [15]. Recently, we found that SND1 serves as a promising malignancy marker and a potential chromatin structure regulator in glioma [16]. Nevertheless, the role of overexpressed SND1 in TNBC metastasis and the related molecular mechanisms still need to be discovered.

CDH1 (E-cadherin) is a transmembrane protein mediating calcium-dependent cell‒cell adhesion [17], which can inhibit tumour cell invasion and limit cell motility [18]. It is well known that CDH1 expression level is tightly related with the occurrence and development among many carcinomas, including colorectal, gastric and ovarian carcinomas [19–21]. In breast cancer, a reduction in CDH1 expression can promote metastasis [22–24]. Consistent with this finding, studies have reported that loss of heterozygosity or transcriptional silencing can downregulate CDH1 and thus, promote tumour metastasis [25–27].

DNMT3A is mainly responsible for de novo methylation of CpG islands in chromatin, and DNMT3A can also methylate hemimethylated DNA [28]. Overexpression of DNMT3A can induce aberrant methylation, oncogene activation and tumour suppressor gene silencing, resulting in genomic instability and oncogenesis [29, 30]. In breast cancer, DNMT3A can silence SOX2, BRCA and HIF-1α [31–33]. Accumulated evidence has emerged to indicate that the DNMT3A level is also linked to the metastasis and poor patients’ survival [33–35]. Nevertheless, the transcriptional regulatory pathway of DNMT3A in TNBC is poorly documented.

In this manuscript, we discovered that SND1 is overexpressed in metastatic TNBC; SND1 upregulation is related to the poor outcome of patients. In addition, we showed that SND1 promotes DNMT3A transcription via its function as a chromatin architectural regulator and that the elevated DNMT3A expression leads to gene methylation and transcriptional inhibition of *CDH1*, which facilitates the metastasis of TNBC cells in vivo and in vitro.

## Methods

### Tissue collection and clinical information

The human tissues of 58 TNBC and 10 glioblastomas (GBM) as positive control were collected from the Tianjin Medical University General Hospital (TMUGH). All the data involving patients were handled under the guidance of the Declaration of Helsinki and the principles of the TMUGH Ethics Committee. The tissues were formalin fixed and paraffin embedded (FFPE). The clinical features of the 58 TNBC from TMUGH and 142 TNBC from The Cancer Genome Atlas (TCGA) (https://cancergenome.nih.gov/) are shown in Additional file 5: Table S1.

### Immunohistochemistry (IHC)

IHC was performed with mouse anti-human SND1 antibody (Santa Cruz sc-166676, dilution ratio 1:25, overnight at 4℃), biotinylated goat anti-mouse secondary antibody (Beyotime A0286, dilution ratio 1:50, incubation for 1 h at room temperature) and HRP-labelled streptavidin (Beyotime A0303, dilution ratio 1:200, incubation for 1 h at room temperature). Finally, the proteins were visualized by diaminobenzidine (DAB) colour development kit (Beyotime P0203). The per cent (labelling index, LI) of SND1 positive cells in 10 fields (× 400) was count under a Leica DM6000B microscope with Image Pro Plus 5.0 software (Media Cybernetics).

### Cell culture and phenotype assay

The HEK293T (ATCC CRL-3216), MDA-MB-231 (ATCC HTB-26) and BT549 (ATCC HTB-122) cell were purchased from the American Type Culture Collection. MDA-MB-231 and BT549 were used for transwell and wound healing assay. The details are listed in the Additional file 6: Methods.

### Plasmids, lentiviruses, and construction of cell lines with stable expression

The details of plasmid construction, lentivirus package and stable cell line transfection were listed in the Additional file 6: Methods.

### DNA electrophoretic mobility shift assay (EMSA)

Additional file 5: Table S4 lists all the probes used in this study. Details are shown in the Additional file 6: Methods.

### cDNA microarray and data analysis

The transcription profiles of MDA-MB-231 cell with different treatments were detected by cDNA microarray (*n* = 3/group). See details in Additional file 6: Methods.

### qRT-PCR and western blot

The qRT-PCR primers were listed in Additional file 5: Table S4; the antibodies used for Western blot were listed in Additional file 5: Table S5. See details in Additional file 6: Methods.

### Chromatin immunoprecipitation (ChIP) and chromosome conformation capture (3C) assay

Scramble and SND1 knockdown TNBCs were used for ChIP and 3C assay; the experimental details are outlined in the Additional file 6: Methods.

### Luciferase reporter assay

The DNMT3A promoter reporter plasmid was established by inserting full length or different fragments of DNMT3A promoter (DNMT3A pro full length/ΔTSS/ΔR1/ΔR2/ΔR1 + R2) into pGL3 vector (Promega, E1751). Then, the aforementioned reporter plasmids were cotransfected with pSG5-SND1 into BT549 and MDA-MB-231 cells via Lipofectamine 3000 (Thermo Fisher L3000015). After 48 h of transfection, the luciferase activities among two breast cancer cells were detected with Dual-Luciferase Reporter Assay Kit (Promega E1910).

### Immunofluorescence (IF)

The expression of CDH1 and DNMT3A in BT549 and MDA-MB-231 was detected by immunofluorescence assay, and the experimental details were outlined in the Additional file 6: Methods.

### Bisulfite sequencing PCR

The bisulfite sequencing PCR assay was used to detected the methylation on the CDH1 gene promoter, and the details were shown in the Additional file 6: Methods.

### Methylcytosine-based DNA immunoprecipitation (MeDIP) for DNA methylation analysis

Genomic DNA was extracted as described above, and 300–500 bp DNA fragments were obtained after sonication. Immunoprecipitation was performed with a mouse monoclonal antibody against 5-methylcytosine (5-mC, Abcam ab10805) and Protein A/G Mix magnetic beads (Millipore LSKMAGAG02). Then, methylated DNA was extracted with DNA extraction reagent (Solarbio D1700-100 T) and analysed by real-time RT‒PCR. All primers were listed at Additional file 5: Table S4.

### Nude mice and tumour cell implantation

MDA-MB-231 expressing firefly luciferase stably (Xenogen, Alameda, CA, USA) was transfected by viruses containing scramble shRNA or either of two different shRNAs targeting SND1. These cells (5 × 10^6^ cells per mouse) were used for tail lateral vein injection pulmonary metastasis model by female SCID mice at six weeks of age. The number of mice in each group was determined by Mead's resource Eq. 200 mg/g D-luciferin (MEM HY-12591A) was injected to the enterocoelia of each mouse for luminescence assay. The autofluorescence images of metastasis tumour were taken by IVIS bioluminescence CCD device (Xenogen), and the image-forming parameters were view field as fifteen cm, binning factor of eight, f-stop as one and exposure time as 120 s. The data were presented by normalized bioluminescence photon flux, which were calculated by background mice without luciferin injection. The Tianjin Medical University Institutional Animal Care Committee approved the entire animal feeding, processing and experimental handing procedures, and confirmed they were in compliance with the ethical regulations.

### Statistical analysis

All data calculation was processed via SPSS version 21.0 (IBM, Chicago, USA). Data were presented as mean ± SD. The significance among means was compared by one-way ANOVA or Student *t* test. The survival time was analysed by Kaplan–Meier (KM) method and compared with log-rank test. The median of protein LI or mRNA log_2_ (fpkm fold) of SND1 was used as the cutoff in survival analyses of the TNBC cohort from TMUGH and TCGA database. The Cox’s proportional hazards regression model was applied for multivariate survival analyses. Statistical significance was assigned at *P < 0.05, **P < 0.01 or ***P < 0.001. All the experiments of cell lines were performed at least in triplicate.

## Results

### TNBC metastasis and poor outcomes were positively correlated with SND1 level of patients

**Fig. 1:**
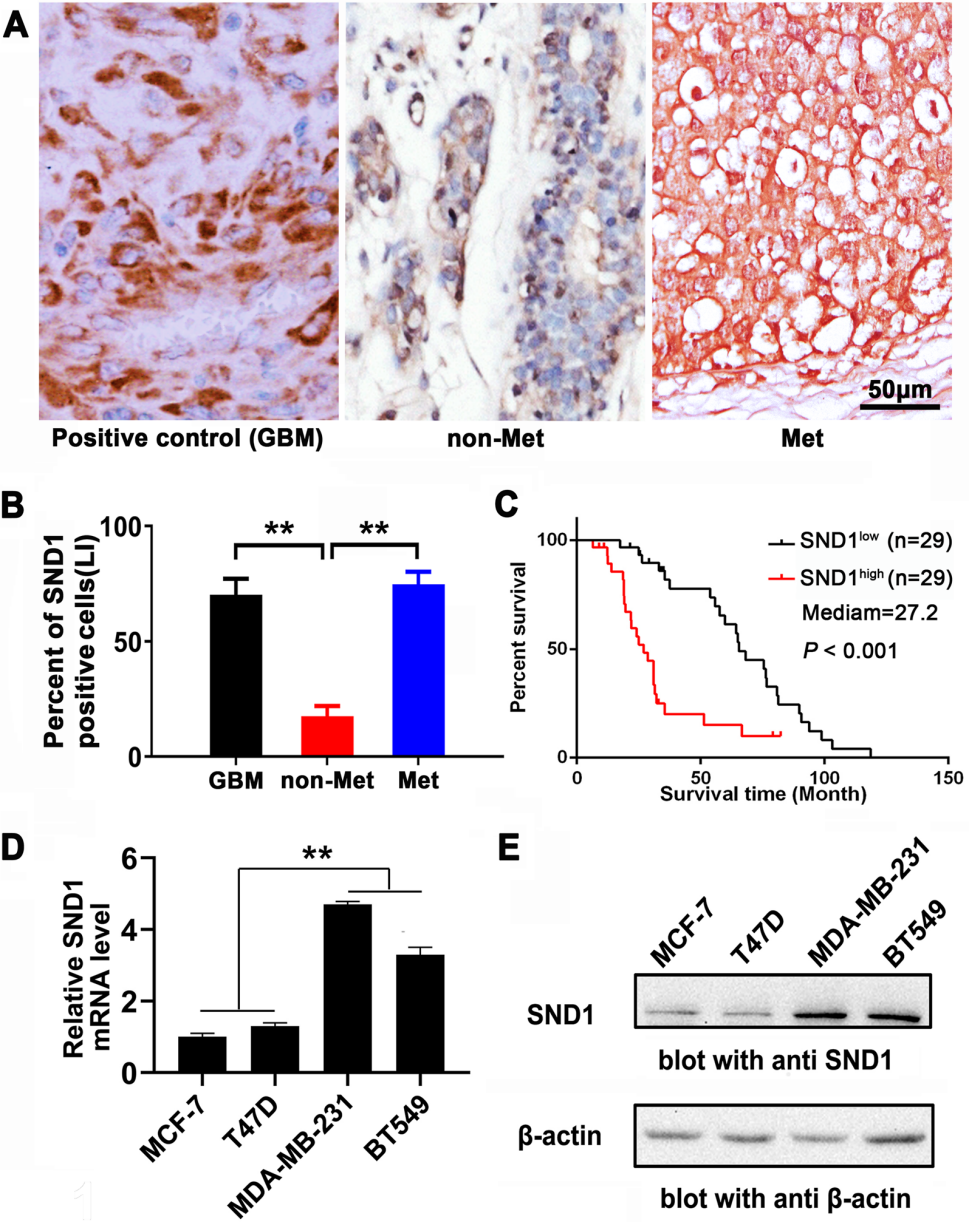
SND1 expression correlates to unfavourable prognosis and metastasis of TNBC. **A** The representative SND1 IHC images of the FFPE samples, 10 case of GBM positive control (GBM), 20 case of TNBC without lymph nodes metastasis (non-Met) and 38 case of TNBC with lymph nodes metastasis (Met). Scale bar: 50 μm. **B** Compare SND1 positive stain per cent among GBM, non-Met and Met groups of the FFPE samples. Under 10 randomly selected 400 × microscopic fields, the SND1 positive stain percentage was calculated (**, *P* < 0.01). **C** K-M survival was plotted based on SND1 level in TNBC patients. Survival in low SND1 subgroup (*n* = 29) showed more favourable prognosis than SND1 highly expression group (*n* = 29; *P* < 0.001). **D** and **E** The protein and mRNA levels of SND1 among four individual breast cancer cells (*n* = 3; **, *P* < 0.01)

The SND1 protein level in TNBC tissues from 38 patients with lymph node metastasis (Met) and 20 patients without metastasis (non-Met) were evaluated by IHC, and 10 glioblastomas (GBM) high-expressing SND1 were used as positive control. The results indicated that the SND1 expression in Met TNBC was significantly higher than that in non-Met TNBC (*P* < 0.01), but the similar as positive control GBM group (*P* > 0.05; Fig. 1A and B). The KM analysis verified that TNBC patients high-expressing SND1 suffered poor outcome (Fig. 1C) and TNBC cohort from TCGA showed similar pattern (Additional file 1: Fig. S1). The multivariate Cox proportional-hazards regression analysis confirmed that SND1 expressive level and TMN stage was the independent predictor for disease-free survival(DFS) and overall survival(OS) of patients with TNBC from both TMUGH and TCGA (Additional file 5: Tables S2 and S3). We also compared the SND1 level among breast cancer cell lines, and the oestrogen receptor-negative MDA-MB-231 and BT549 cells had stronger SND1 expression than luminal-like cell lines (Fig. 1D, E).

### SND1 promotes the invasion and migration of TNBC cells

**Fig. 2:**
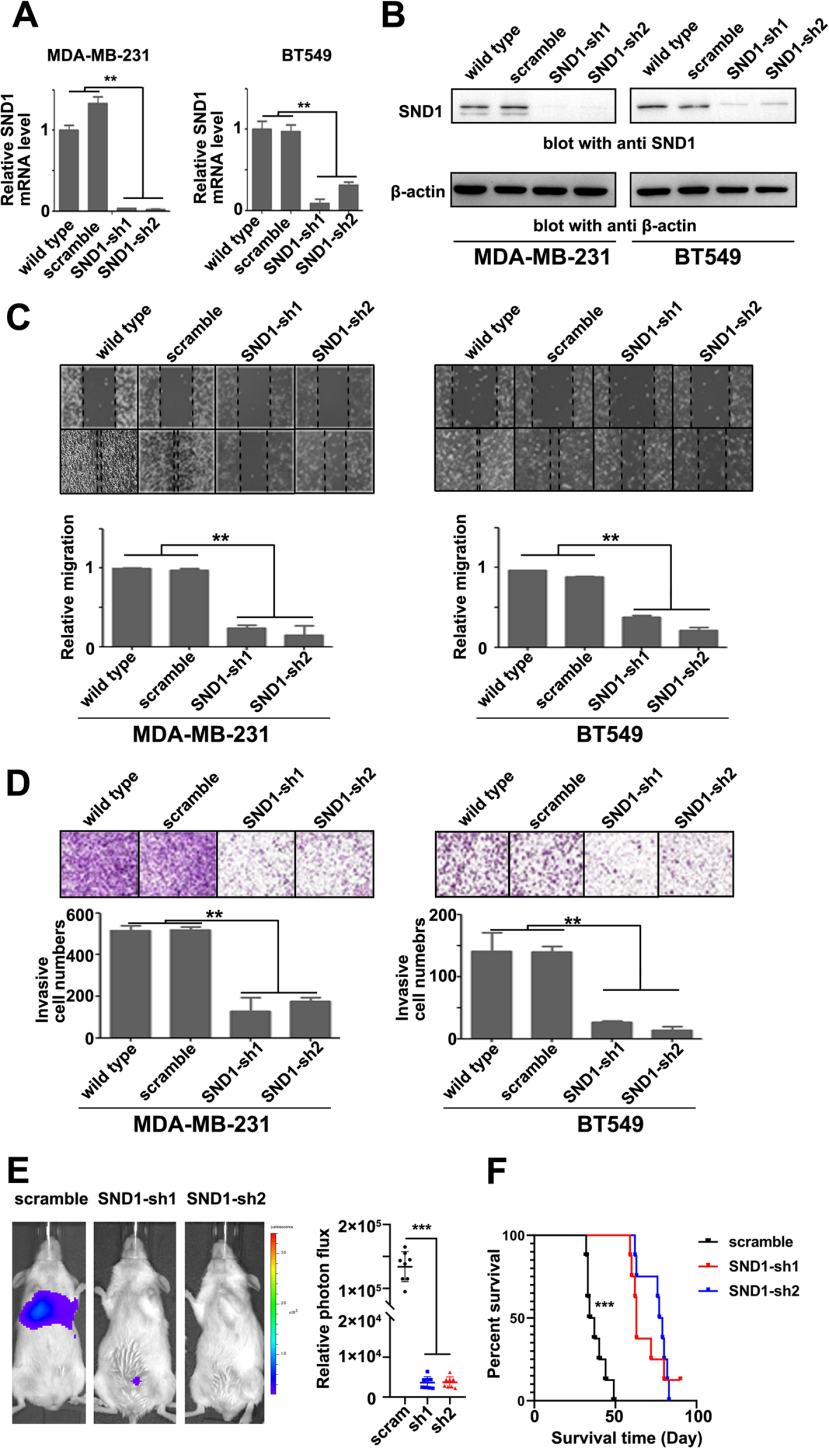
SND1 promotes TNBC cell migration and invasion. **A**, **B** qRT-PCR and Western blotting results of SND1 levels in MDA-MB-231 and BT549 cells of wild type control (wild type), scramble control (scramble), SND1 knocking down (SND1-sh1; SND1-sh2; *n* = 3; **, *P* < 0.01). **C** The wound healing assay results of aforementioned breast cancer cells (*n* = 3; **, *P* < 0.01). **D** The transwell assay results of above-mentioned cells (*n* = 3; **, *P* < 0.01). **E** MDA-MB-231 cells stably expressing scramble shRNA (scramble) or SND1 shRNAs (SND1-sh1, sh2) were injected into caudal vein of SCID mice. The pulmonary metastasis luminous signals were measured by luminous CCD system (*n* = 8; ***, *P* < 0.001). **F** K-M survival analysis showed that mice inoculated with SND1knocking down cells (sh1/sh2) had longer survival time than the control mice (scramble, ***, *P* < 0.001)

To detect the role of SND1 on TNBC migration and invasion, we used BT549 and MDA-MB-231 cell lines as models for wound healing, Transwell and establishment of a tail vein injection inducing pulmonary metastasis model in mice. Both qPCR and Western blot were performed to double check the knockdown efficiency of SND1 (with SND1-sh1 and SND1-sh2) in two breast cancer cell lines (Fig. 2A, B). The wound healing assay indicated the migration of MDA-MB-231 and BT549 cells was diminished after knockdown of SND1 (Fig. 2C). The Transwell results also confirmed that SND1 knockdown blocked the invasion of aforementioned cells (Fig. 2D). Such results indicated that SND1 might promote the metastatic phenotype of TNBC cells in vitro.

To confirm the importance of SND1 to in vivo TNBC cell metastasis, MDA-MB-231 cells with stable luciferase expression were infected by lentiviruses with control RNA (scramble) or an SND1 shRNA (sh1 or sh2). The aforementioned cells were intravenously injected to the tail veins of SCID mice (*n* = 8). Bioluminescence imaging was generated via an IVIS luminescence CCD device (Xenogen) to quantify the lung metastasis of ectopically implanted TNBC cells every week. The results demonstrated that, compared to the scramble subclones, the SND1-sh1 and SND1-sh2 cells had a significantly decreased metastatic capacity (Fig. 2E). The Kaplan–Meier analysis data indicated that survival times of mice from SND1 knockdown group (SND1-sh1/sh2) were extended comparing to the scramble group (Fig. 2F). Western blot was perform with the pulmonary metastatic tissues in order to detect SND1 expression and guarantee the implementation of follow-up experiments (Additional file 2: Fig. S2). The above results indicate that SND1 may play an important role in breast cancer metastasis and predict worse prognosis of TNBC.

### SND1 regulates the expression of genes that are relevant to TNBC biology

**Fig. 3:**
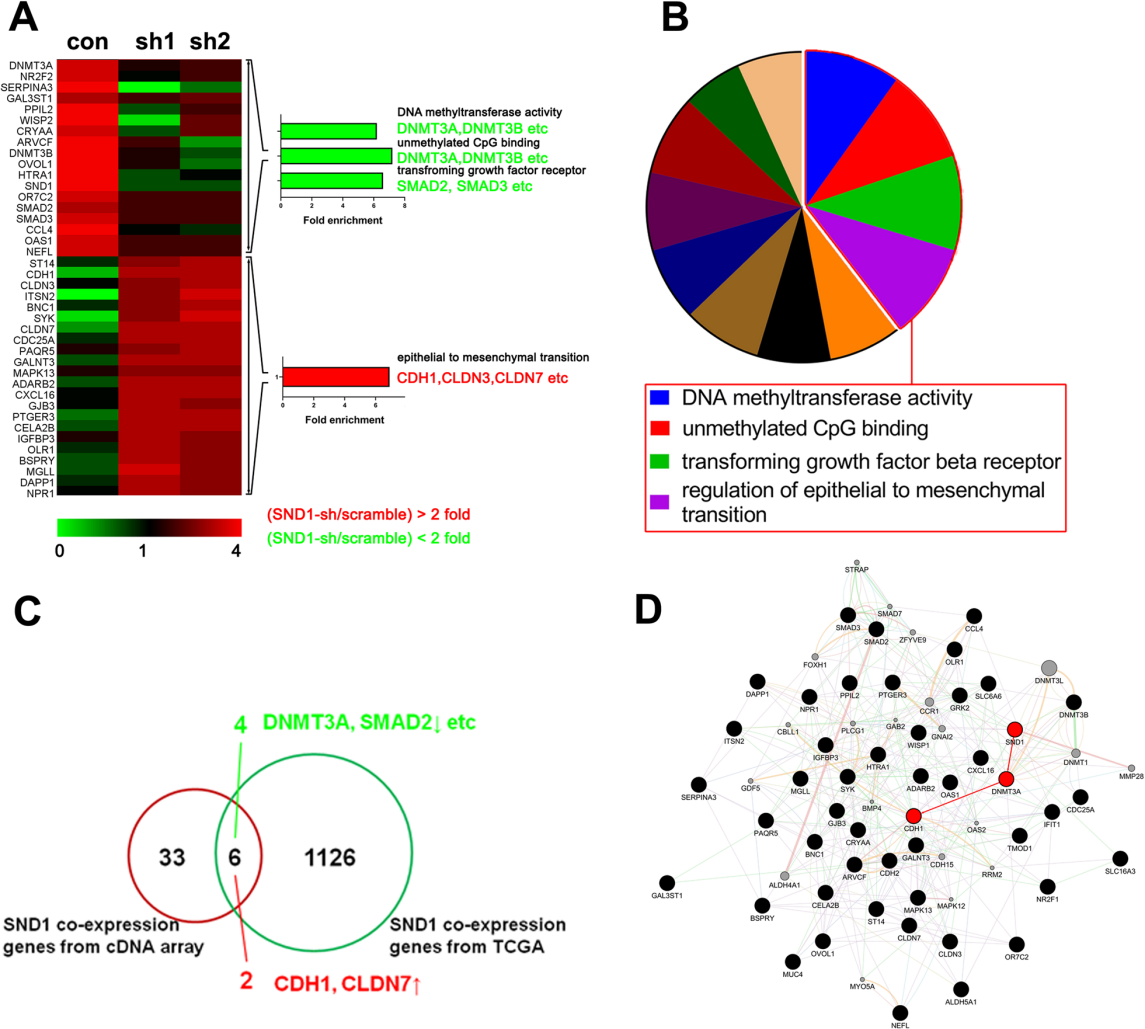
SND1 knockdown regulates a group of DNA methylation and cell motility genes in TNBC. **A** Hierarchical clustering of scrambled shRNAs (Scramble) or SND1 shRNAs (SND1-sh1/sh2) transfection were shown. After SND1 knocking down, 17 downregulated and 22 upregulated genes were screened out and annotated (green, downregulated gene; red, upregulated gene). **B** The KEGG enriched pathways among the SND1 potential regulatory targets were listed at the red box. **C** Venn plot showed SND1 downstream target genes. **D** Network plot descripting relationships among SND1, DNMT3A and CDH1 (bold red circle) and other related pathway members (bold black circle)

To clarify the detail mechanism by which SND1 promotes TNBC metastasis, we identified SND1 downstream genes via cDNA microarray assay in MDA-MB-231 cells. After SND1 knockdown, the expressions of 17 genes were decreased and 22 genes were increased (Additional file 5: Table S6). Among the 39 potential SND1 downstream genes, *DNMT3A*, *DNMT3B*, *SMAD2* and *SMAD3* were the most downregulated, and *CDH1*, *CLDN*3 and *CLDN7* were the most upregulated (Fig. 3A, data are presented as the means of each group, *n* = 3, two way fold > 2, FDR < 0.05). Gene Ontology analysis showed that the potential downstream genes were involved in DNA methyltransferase activity, unmethylated CpG binding, TGF-β receptor signalling and the regulation of epithelial-to-mesenchymal transition (Fig. 3B). Through Pearson correlation analysis of gene expression data in the TCGA database, 1132 genes coexpressed with SND1 were selected (*P* < 0.05, R > 0.3). By overlapping these genes with the 39 genes identified in the above cDNA chip analysis, it was found that there were 4 direct target candidate genes (green) and 2 indirect target candidates (red). Across all the candidates, *DNMT3A* had the strongest positive relevance to *SND1* expression, and *CDH1* level was the most negatively related to that of *SND1* (Fig. 3C). Then, the Cytoscape GeneMANIA plug-in was used to analyse and create the SND1 regulatory schematic diagram (Fig. 3D). These results suggested SND1 may regulate CDH1 expression by promoting *DNMT3A* transcription.

### SND1 promotes the transcription of DNMT3A in TNBC cells

**Fig. 4:**
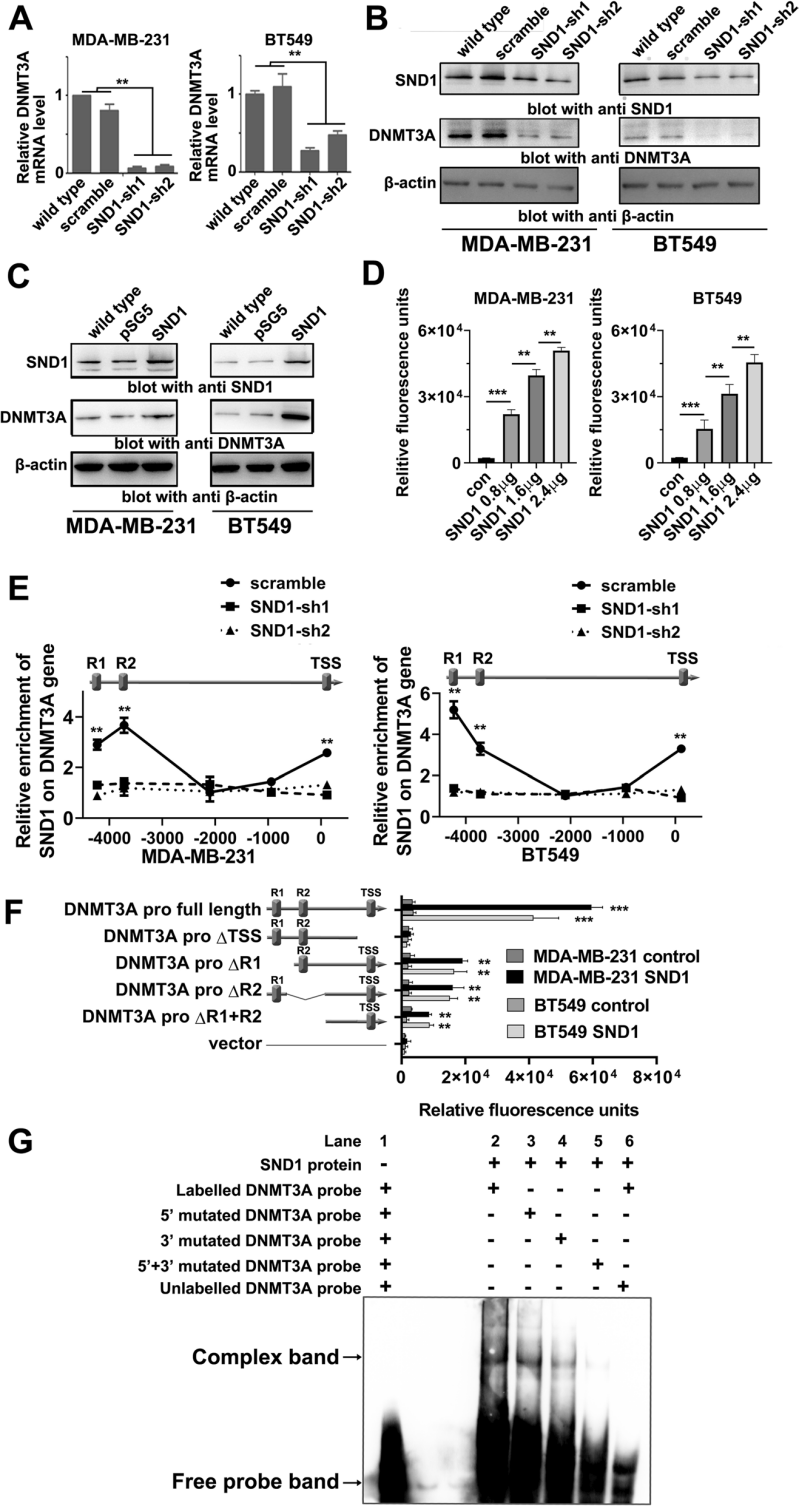
SND1 promotes the transcription of DNMT3A in TNBC cells. **A** qRT-PCR results of DNMT3A levels in wild type MDA-MB-231 and BT549 (wild type), scramble shRNA bearing cells (scramble) and two SND1-knocking down shRNA transfecting cells (SND1-sh1/-sh2; *n* = 3; **, *P* < 0.01). **B** The SND1 and DNMT3A western results in aforementioned MDA-MB-231 and BT549 cells. **C** Western blot results of SND1 and DNMT3A levels in two breast cancer cells of wild type control (wild type), empty vector control (pSG5) and SND1 overexpression (SND1). **D** Luciferase assay results of DNMT3A transcription activity in BT549 and MDA-MB-231 cells of dosage dependent of SND1 level. **E** ChIP-PCR assay results of the interaction pattern of SND1 with conserve sites at DNMT3A promoter in MDA-MB-231 and BT549 cells of scramble control (scramble) and SND1 knocking down (SND1-sh1; SND1-sh2; *n* = 3; **, *P* < 0.01). **F** Luciferase reporter assays result of MDA-MB-231 and BT549 cells via the pGL3 vector inserting with different DNMT3A promoter fragments (full-length promoter, full length; deletion of TSS site, ΔTSS; deletion of R1/R2 site, ΔR1/R2; deletion of both R1 and R2, ΔR1 + R2; empty vector, vector). The empty expression vector pSG5 groups were set as references (control), and SND1 overexpression groups were used to detected DNMT3A transcription enhanced by SND1 (SND1; *n* = 3; **, *P* < 0.01; *n* = 3; ***, *P* < 0.001). **G** Interaction among in vitro translated SND1 and DNMT3A promoter verified by EMSA (Lanes 2, 3 and 4)

Since bioinformatics analysis showed that *DNMT3A* was the putative candidate *SND1* downstream gene, we further examined the relationship between *DNMT3A* and *SND1*. The qRT‒PCR and Western blot results showed expression levels of DNMT3A were dramatically decreased in MDA-MB-231 SND1-sh1/2 and BT-549 SND1-sh1/2 cells (Fig. 4A, B). Overexpression of SND1 up-regulated the expression of DNMT3A in both TNBC cells (Fig. 4C). The above results indicated that SND1 might promote DNMT3A expression via transcriptional activation. Then, a luciferase reporter assay was adopted to explore whether *DNMT3A* transcription is directly regulated by SND1. The promoter sequence of *DNMT3A* was inserted into pGL3 reporter plasmid to construct the pGL3-DNMT3A pro reporter vector. Then, the reporter vector was cotransfected into BT549 and MDA-MB-231 cells with pSG5-SND1 or pSG5-con. The relative luciferase activity was significantly increased in all SND1 overexpression groups, and the luciferase signals were positively correlated with the SND1 expression level (Fig. 4D).

Bioinformatics analysis indicated three individual SND1 interaction positions on the *DNMT3A* promoter (Fig. 4E, upper part); one was located at the transcription start site (TSS), and the other two were located upstream of the TSS (R1 and R2). The ChIP assay showed SND1 protein could bind at R1, R2 and TSS in both tested TNBC cell lines and that knockdown of SND1 attenuated the interaction of SND1 with all three conserved sites, further confirming the specificity for the recruitment of SND1 on *DNMT3A* promoter (Fig. 4E). The role of SND1 enrichment at conserved sites in the transcription of *DNMT3A* was further determined by dual-luciferase reporter assay. Deletion of TSS completely blocked the inducing effect of SND1 on *DNMT3A* transcription, and deletion of R1, R2 or both R1 and R2 only partially inhibited the transcriptional activation of *DNMT3A* induced by SND1 (Fig. 4F). The aforementioned data showed that the recruitment of SND1 on TSS is essential for transcriptional activation of *DNMT3A* and that the interaction between SND1 and R1 or R2 can enhance *DNMT3A* transcription. R1, R2 and TSS have a universal conserved motif, 5’-TGATCTCGGCTCACT-3’, which might be the SND1 recognition site. To confirm the detailed interaction mechanism of SND1 with these different sites, in vitro translated SND1 protein was purified and subjected to EMSA (Fig. 4G). SND1 formed complexes with labelled probes containing the conserved motif (Fig. 4G, Lane 2), mutation of the 5’ end, the 3’ end, or both the 5’ and 3’ ends of the conserved motif inhibited the binding of SND1 (Fig. 4G, Lanes 3, 4 and 5), and the unlabelled cold probe serving as the negative control also competitively inhibited the formation of the band corresponding to the complex (Fig. 4G, Lane 6). Such results indicated that SND1 interacts with *DNMT3A* promoter by recognizing the conserved motif in R1, R2 and TSS.

### SND1 promotes *DNMT3A* transcription via mediating long-range chromatin remodelling on *DNMT3A *promoter in TNBC cells

**Fig. 5:**
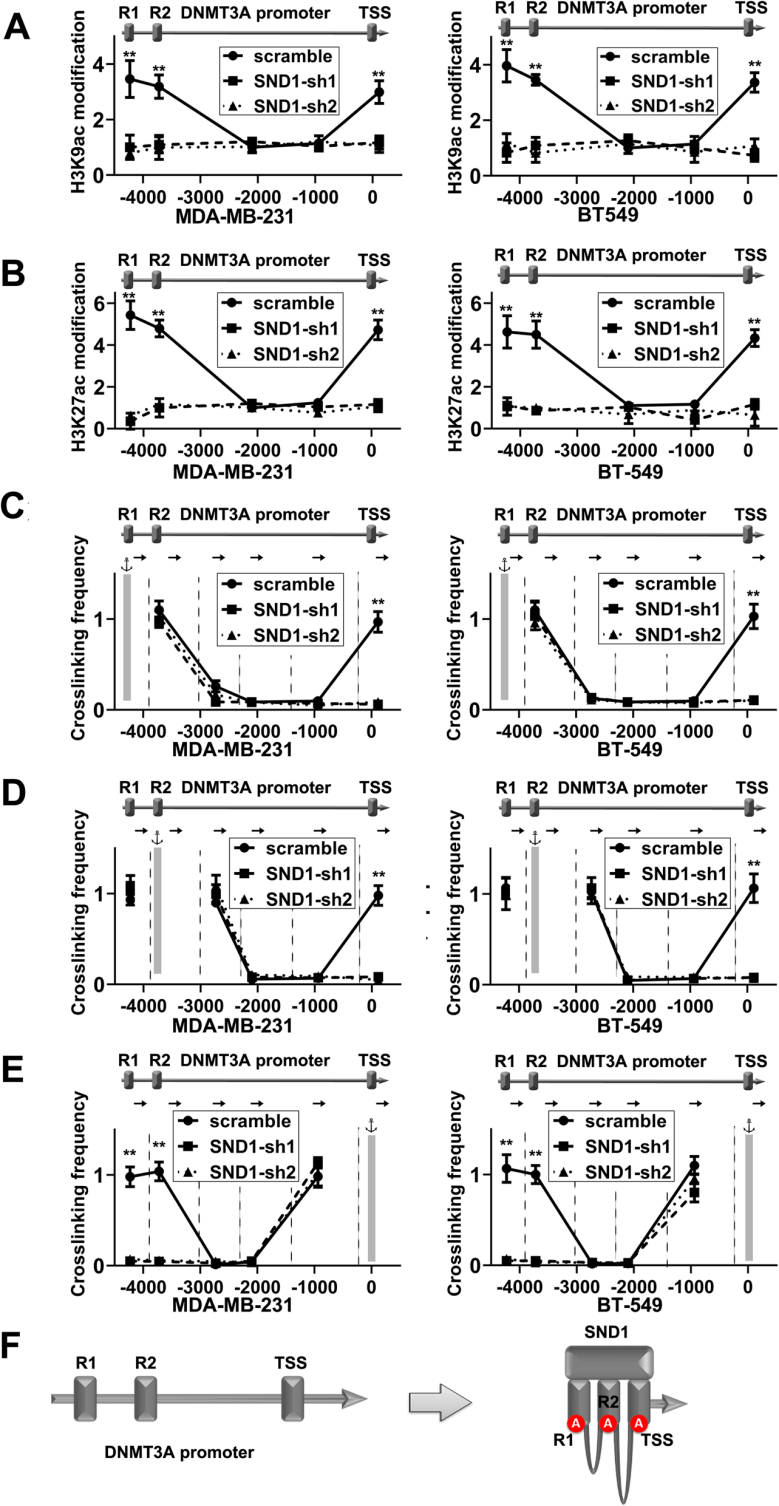
SND1 enhances transcription of DNMT3A by promoting chromatin long-range interaction in DNMT3A prompter. **A**, **B** ChIP assay data showed the histone acetylation on H3K9ac (**A**) and H3K27ac (**B**) at DNMT3A promoter after SND1 knocking down. **C** The first anchor was SND1 recognition regions R1, 3C data showed the DNA cross-link among R1 to R2 and the TSS without SND1 knockdown. The SND1 depletion inhibited the forming of R1/TSS cross-link but not the forming of R1/R2 cross-link. The vertical lines displayed the restriction enzyme cleavage sites and the arrows represented PCR primers. **D** The second anchor was R2, and the cross-links among R2 to R1 and TSS were displayed by 3C. **E** TSS was the final anchor, and the cross-links among TSS to R1 and R2 were shown. **F** The image-schematic structure presented the mechanism of SND1 promoting DNMT3A transcription. All data were shown in mean ± SD. *n* = 3, **, *P* < 0.01

Chromatin histone acetylation is an essential event in initiating long-range chromatin architectural alterations and inducing gene transcription. Our previous results showed that histone acetyltransferases can be recruited by SND1 to certain gene loci and lead to chromatin conformation rearrangement and gene transcription. ChIP‒qPCR targeting histone H3K9Ac and H3K27Ac showed that the histone acetylation occurred at the same position of SND1 binding regions in TNBC cells (Fig. 5A, B; scramble; R1, R2 and TSS) and that silencing of endogenous SND1 decreased the H3K9Ac and H3K27Ac levels in both TNBC cell lines (Fig. 5A, B; SND1-sh1/sh2; R1, R2 and TSS). The histone acetylation induced by SND1 unlocks the potential for chromatin to be remodelled to another conformation.

To assemble the transcription factors and the distant cis-transcription elements that occupied by them, topological alterations in chromatin are necessary. 3C analysis was applied to determine the stereoscopic proximity of the conserved motif in R1, R2 and TSS. When the R1 site was set for the anchor of PCR, strong chromatin cross-linking signals were detected at R1, R2 and TSS in TNBC cells, while SND1 knockdown blocked only the long-range association between R1 and TSS (Fig. 5C). When R2 region was set as anchor, a similar cross-linking signal between R2 and TSS was detected (Fig. 5D). When TSS was set as anchor, TSS region could interact with both R1 and R2 (Fig. 5E). SND1 could recruits to the histone acetylation-modified R1, R2 and TSS and induce chromatin conformation remodelling, which finally promote DNMT3A transcription (Fig. 5F).

### SND1 represses CDH1 expressionviaDNMT3A-induced promoter methylation

**Fig. 6:**
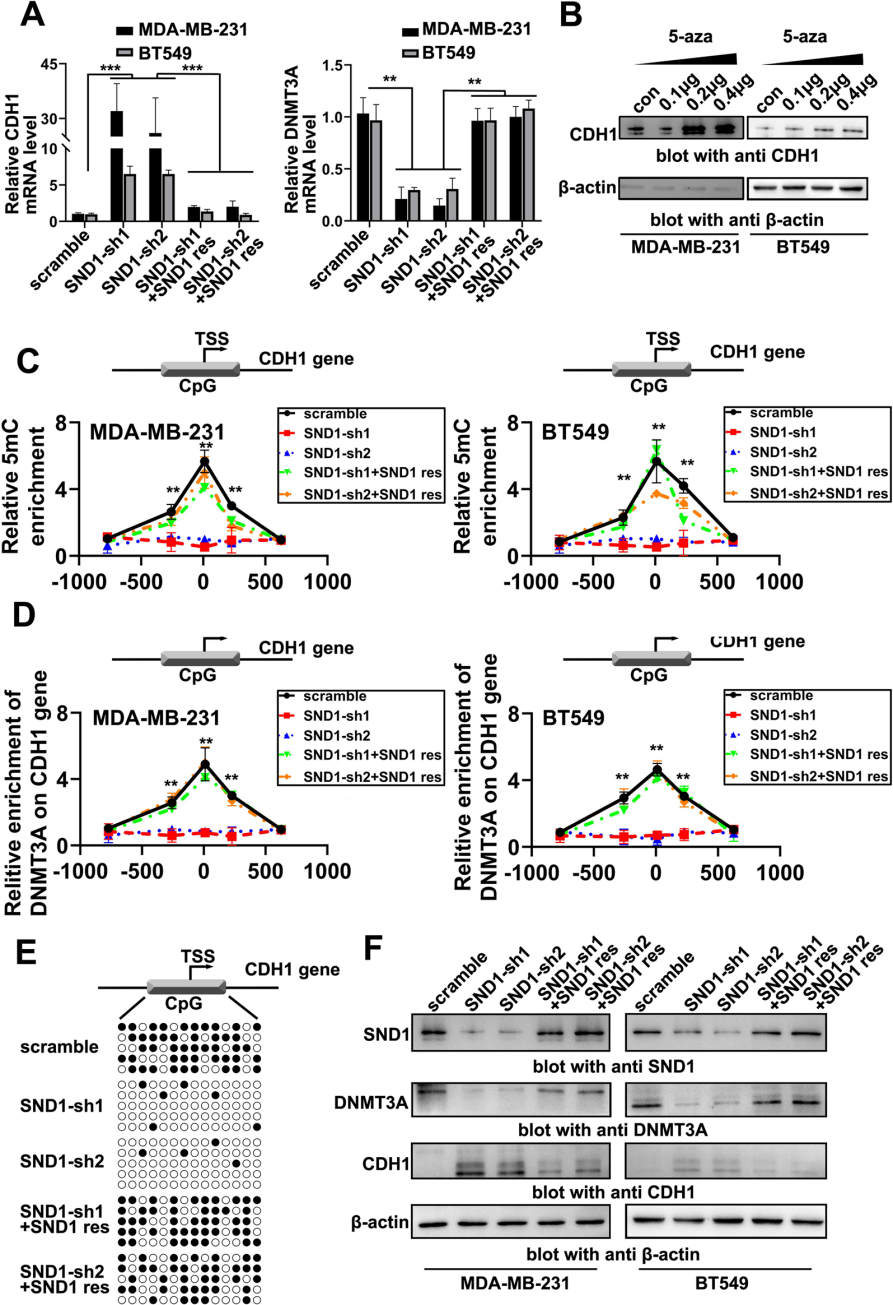
SND1 represses the expression of CDH1 via inducing CDH1 promoter methylation. **A** qRT-PCR results of CDH1 and DNMT3A levels in BT549 and MDA-MB-231 cells of scramble group (scramble), two SND1-knocking down groups (SND1-sh1; SND1-sh2) and two SND1 rescue expression groups (SND1-sh1 + SND1 res; SND1-sh2 + SND1 res; *n* = 3; **, *P* < 0.01, ***, *P* < 0.001). **B** CDH1 Western blotting data from MDA-MB-231 and BT549 cell treated with different amounts of 5-aza. **C** MeDIP results of methylation level on CHD1 promoter in aforementioned treated BT549 and MDA-MB-231 cells (scramble; SND1-sh1; SND1-sh2; SND1-sh1 + SND1 res; SND1-sh2 + SND1 res; *n* = 3; **, *P* < 0.01). **D** ChIP results of DNMT3A binding condition on CDH1 promoter in aforementioned MDA-MB-231 and BT549 cells (*n* = 3; **, *P* < 0.01). **E** Bisulfite sequencing PCR results of methylation level on CHD1 promoter in aforementioned MDA-MB-231 and BT549 cells. **F** Western blotting data of SND1, DNMT3A and CDH1 levels in aforementioned MDA-MB-231 and BT549 cells

The microarray data showed that SND1 expression was also negatively correlated with that of the key metastasis factor CDH1 in TNBC cells (Fig. 3A). CDH1 is a potential DNMT3A target. Therefore, we speculated that SND1 could inhibit CDH1 expression by upregulating DNMT3A and then, in turn promote TNBC cell invasion and migration. To verify the above-mentioned hypothesis, we first measured the expression of CHD1 and DNMT3A in MDA-MB-231 and BT549 cells. As expected, we found that SND1 knockdown caused a decrease in *DNMT3A* mRNA level and an increase in CDH1 (Fig. 6A; SND1-sh1/sh2), while restoration of SND1 expression reversed these changes in DNMT3A and CDH1 in SND1 knockdown cells (Fig. 6A; SND1-sh1/sh2 + SND1 res). The universal DNA methylation inhibitor 5-azacytidine (5-aza) dose-dependently repressed the expression of CDH1, indicating CDH1 should be one of the downstream targets of DNMT3A in TNBC cells (Fig. 6B). The methylated DNA immunoprecipitation (MeDIP) results showed that the CpG island at the promoter of *CDH1* was highly methylated in scramble control TNBC cells (Fig. 6C; scramble). SND1 knockdown inhibited the methylation of the *CDH1* CpG island (Fig. 6C; SND1-sh1/sh2), while SND1 reconstitution by transfection restored this methylation (Fig. 6C; SND1-sh1/sh2 + SND1 res). DNMT3A needs to be recruited to certain loci of CpG islands to catalyse DNA methylation. The ChIP assay results showed that DNMT3A was recruited at the CpG Island on promoter of *CDH1* to maintain its methylation level (Fig. 6D; scramble). SND1 knockdown led to dissociation of DNMT3A from the *CDH1* promoter (Fig. 6D; SND1-sh1/sh2), while SND1 reconstitution by transfection restored the recruitment of DNMT3A to the *CDH1* promoter (Fig. 6D; SND1-sh1/sh2 + SND1 res). The bisulfite sequencing PCR results showed a similar pattern as the MeDIP and ChIP results. Of the 14 potential CpG methylation sites, most were demethylated after SND1 knockdown, and those sites were remethylated after SND1 reconstitution by transfection (Fig. 6E). The Western blotting results further demonstrated SND1 knocking down led to the depletion of DNMT3A and appearance of CDH1, while reconstitution by transfection of SND1 reversed these changes in DNMT3A and CDH1 expression in SND1 knockdown cell lines (Fig. 6F). These above results confirmed that SND1 can promote DNMT3A expression, inducing CDH1 promoter methylation and finally inhibiting CDH1 expression.

**Fig. 7:**
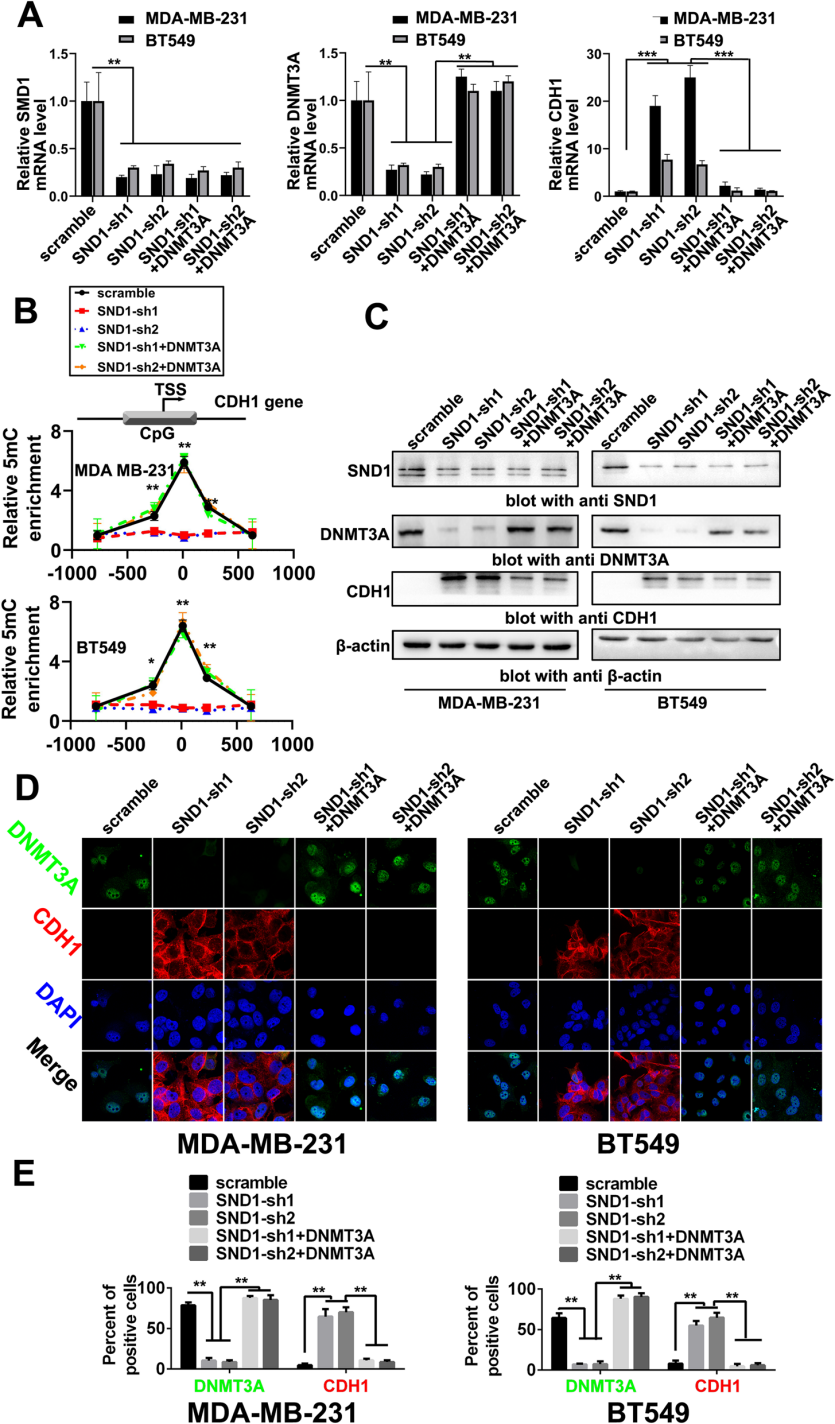
DNMT3A promotes methylation of CDH1 promoter and inhibits CDH1 expression. **A** PCR tests expression of SND1, DNMT3A and CDH1 in BT549 and MDA-MB-231 of scramble shRNA (scramble), two SND1 knocking down (SND1-sh1/sh2) and two DNMT3A rescue expression (SND1-sh1 + DNMT3A; SND1-sh2 + DNMT3A res; *n* = 3; **, *P* < 0.01, ***, *P* < 0.001). **B** MeDIP analysis of methylation level on CHD1 promoter in aforementioned BT549 and MDA-MB-231 cells (*n* = 3; *, *P* < 0.05, **, *P* < 0.01). **C** Western results of SND1, DNMT3A and CDH1 expressions in aforementioned MDA-MB-231 and BT549 cell. **D** Immunofluorescence results of DNMT3A and CDH1 expressions in aforementioned MDA-MB-231 and BT549 cell. (E) Compare positive stain per cent of DNMT3A and CDH1 in aforementioned MDA-MB-231 and BT549 cell (*n* = 5; **, *P* < 0.01)

Then, we used DNMT3A reconstitution by transfection to further confirm that DNMT3A is the key mediator of SND1-induced CDH1 *promoter* methylation. Subsequently, BT549 and MDA-MB-231 cell lines were constitutively transduced with scrambled shRNA (scramble; control), constitutively transduced with SND1-shRNA (SND1-sh1/sh2; for endogenous SND1 knockdown), or subjected to SND1-shRNA/DNMT3A co-expression (SND1-sh1/sh2 + DNMT3A; rescue of DNMT3A expression). As expected, we found that rescue of DNMT3A expression completely blocked the *CDH1* mRNA transcription induced by SND1 knockdown (Fig. 7A). Compared to the demethylated *CDH1* promoter in SND1 knockdown cells, reconstitution of DNMT3A also catalysed the remethylation of the *CDH1* promoter to the basal levels measured in scramble control TNBC cells (Fig. 7B). Western blotting further confirmed that reconstitution of DNMT3A in SND1 knockdown cells reduced CDH1 expression to the same level as that in scramble control TNBC cells (Fig. 7C). The immunofluorescence (IF) results revealed a similar pattern as the above Western blot results (Fig. 7D); SND1 knockdown inhibited DNMT3A expression and increased CDH1 expression. After reconstitution of DNMT3A in SND1-sh1/sh2 cells, the CDH1 protein level was decreased back to basal level measured in scramble group (Fig. 7E).

### SND1 promotes the metastatic phenotype of TNBC cellsviaDNMT3A

**Fig. 8:**
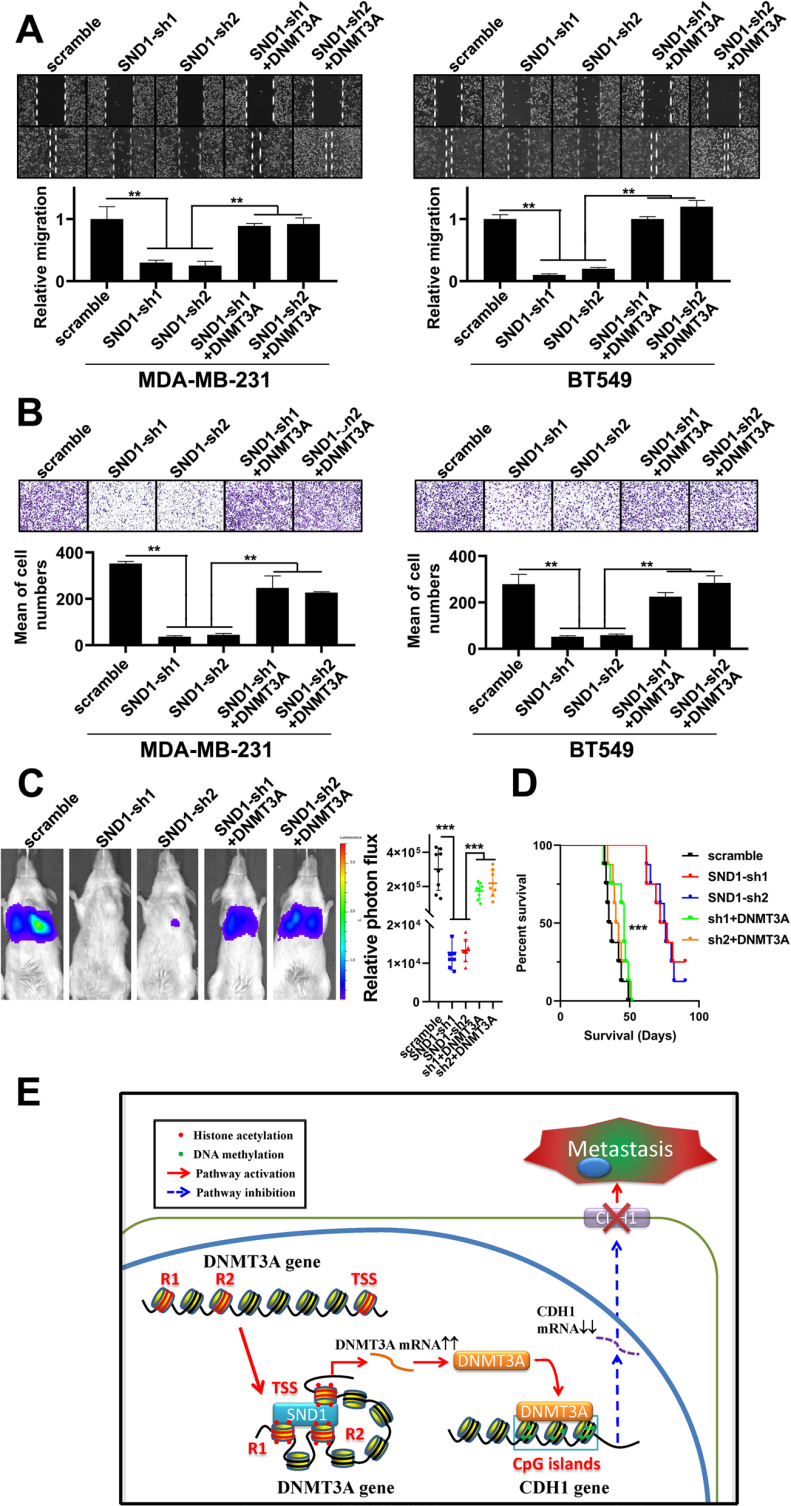
SND1 promotes the metastatic phenotype of TNBC cells via DNMT3A. **A** Scratch wound assay results from MDA-MB-231 and BT549 cells of scramble shRNA (scramble), two SND1 knocking down (SND1-sh1; SND1-sh2) and two DNMT3A rescue expression (SND1-sh1 + DNMT3A; SND1-sh2 + DNMT3A res; *n* = 3; **, *P* < 0.01). **B** Transwell results of aforementioned BT549 and MDA-MB-231 cells (*n* = 3; **, *P* < 0.01). **C** Tumour cell pulmonary metastasis assay results showed the metastasis potential of MDA-MB-231 transfected with scramble control (scramble), SND1-knockdown (SND1-sh1; SND1-sh2), DNMT3A rescue expression (SND1-sh1 + DNMT3A; SND1-sh2 + DNMT3A). Representative bioluminescence images of pulmonary metastasis were presented (left part). The relative optical intensities of photon flux were calculated (right part; *n* = 8; ***, *P* < 0.001). **D** K-M assay results from that different groups of mice bearing MDA-MB-231 cells of scramble control (scramble), SND1-knockdown (SND1-sh1; SND1-sh2), DNMT3A rescue expression (SND1-sh1 + DNMT3A; SND1-sh2 + DNMT3A; *n* = 8; ***, *P* < 0.001). **E** Schematic diagram of SND1 function on DNMT3A and CDH1

To identify the effect of DNMT3A to SND1-induced TNBC malignancy in vitro, we evaluated the metastatic phenotype of the above-mentioned TNBC cells used to generate the data in Fig. 7 by transwell and wound healing assays. The migration ability of MDA-MB-231 and BT459 cells was significantly decreased in the SND1-sh1/sh2 groups, while cell migration in the SND1-sh1/sh2 + DNMT3A groups was maintained at the normal level observed in scramble control cells (Fig. 8A). The Transwell assay results revealed a similar pattern as the wound healing assay results, and DNMT3A reconstitution by transfection abolished the inhibition of TNBC cell invasion, consistent with SND1 knockdown effects (Fig. 8B).

We established an experimental model of pulmonary metastasis via tail vein injection to further investigate whether DNMT3A is the key mediator of SND1-induced in vivo metastasis of TNBC cells. MDA-MB-231 cells with SND1 knockdown or DNMT3A reconstitution (scramble, SND1-sh1/sh2, SND1-sh1/sh2 + DNMT3A) were infected with lentivirus expressing firefly luciferase. Bioluminescence imaging of MDA-MB-231 cells confirmed that SND1 knocking down decreased the pulmonary tumour signal significantly, whereas restoration of the expression of DNMT3A would neutralized the aforementioned effect of SND1 knockdown (Fig. 8C). The Kaplan–Meier analysis results indicated that mice in the SND1 knocking down groups (SND1-sh1, sh2) had longer survival time than those in the scramble control group (scramble), while mice in the DNMT3A rescue group (SND1-sh1/sh2 + DNMT3A) had similar life span as those of mice in the scramble control groups (Fig. 8D). In summary, SND1 promotes DNMT3A expression via inducing chromatin architecture rearrangement, DNMT3A supresses CDH1 and promotes invasion and migration of TNBC cells. DNMT3A is core mediator through the SND1/DNMT3A/CDH1 axis which SND1 promotes TNBC cell metastasis (Fig. 8E).

## Discussion

SND1 modulates downstream genes via different pathways. SND1 can promote colon cancer cells proliferation by suppressing APC and activating telomerase [11, 36]. SND1 can also induce hepatic tumour-initiating cells forming via regulating Akt signalling [37]. In addition, SND1 enhances angiogenesis by activating NF-KB and inducing the expression of miR-221 [38]. SND1 regulates the error mitosis correction and DTX chemoresistance in prostate cancer cells [39].

Accumulated evidence has shown that SND1 might be a potential chromatin architectural regulator [40]. Our recent study showed SND1 can induce histone lysine acetylation in breast cancer via GCN5 [15]. In glioma cells, SND1 can induce a long-range chromatin interaction loop between two SND1 recognition positions on the *RHOA* promoter [16]. In this study, we discovered that SND1 induces the transcription of DNA methyltransferase 3A (DNMT3A) in breast cancer cells. SND1 enhanced histone acetylation at three individual sites in the DNMT3A promoter and then, promoted the formation of double loops among the three sites. The above results linked the histone acetylation induced by SND1 with a unique chromatin conformation remodelling event, in which SND1 acts as a stereoscopic chromatin conformation modulator that promotes gene expression via regulating multiple long-range interactions between transcriptional cis-acting elements.

The transfer of a methyl group to a cytosine in a CpG island to form 5-methylcytosine (5mC) is the most general process of DNA methylation. Aberrancy of the inheritable epigenetic process in the promoters of essential proliferation control genes, such as tumour suppressors, is milestone event during breast cancer tumorigenesis [41]. Compared with other breast cancer subtypes, TNBC cancer shows extensive promoter hypermethylation in key oncogenic genes, indicating that targeting the methylation machinery in TNBC cells may have clinical benefits [42]. DNMT3A acts as a methyltransferase for de novo methylation and maintains 5-methylcytosine modification at CpG sites. Accumulating evidence indicates that DNA hypomethylation resulting from DNMT3A activity is significantly related to chromatin remodelling and critically participates in the tumorigenesis and malignance of breast cancer [43]. Breast cancer patients with advanced clinical stage disease or distant metastasis often have high expression of DNMT3A. Moreover, DNMT3A expression has been correlated with shorter DFS and OS times in breast cancer patients [33]. Nevertheless, the downstream and upstream pathways underlying DNMT3A-related TNBC metastasis still need to be further clarified. In present research, we found that SND1 can directly upregulate DNMT3A transcription via modulating long-distance chromatin looping in the promoter of DNMT3A. The abnormally expressed DNMT3A promotes the hypermethylation and transcriptional inhibition of the *CDH1* gene.

CDH1 can mediate connections between cancer cells, and loss or deficiency of CDH1 expression weakens cell‒cell adhesion, facilitating the dissociation of cells from surrounding tissues [23, 44]. Deficiency of CDH1 is closely related to breast cancer metastasis [45]. Loss of CDH1 is also strongly related to breast cancer progression in patients [46]. Our study observed that the high level expressed SND1 in TNBC cell lines significantly increased the expression of DNMT3A, leading to aberrant methylation patterns of CDH1 and promoting breast cancer metastasis.

## Conclusions

In summary, we discovered novel mechanisms about migration and invasion activities driven by SND1 via directly promoting *DNMT3A* transcription through induction of histone acetylation and remodelling of the stereo chromatin structure of the *DNMT3A* promoter. The upregulated DNMT3A increased the methylation level at the *CDH1* gene locus and suppressed its expression, leading to TNBC invasion and migration (Fig. 8E). Our results provide a potential rationale for targeting SND1 and DNMT3A in TNBC therapy. Furthermore, our study connected SND1-induced long-distance chromatin remodelling with metastasis and bleak prognosis of TNBC patients.

### Supplementary Information

**Additional file 1. Supplementary Fig. S1.** SND1 expression correlates to lymphatic metastasis and patient survival of TNBC. (A) Compare SND1 expression level between patients with (Met) or without (non Met) lymphatic metastasis from the TCGA TNBC (*, *P* = 0.016, n = 142). (B) K-M survival was plotted based on SND1 level in TNBC patients of TCGA. The low SND1 subgroup (n=71) showed more favourable prognosis than SND1 highly expression group (n = 71; *P* = 0.029).

**Additional file 2. Supplementary Fig. S2.** The metastasis tumour samples from mice were used to validate the expressions of SND1 and SND1 targets. Western results of SND1, CDH1, CLDN3 and CLDN7 expressions in metastasis tumour samples from mice transplanted with MDA-MB-231 cells of scramble control (scramble; n=8) or SND1 knocking down (SND1-sh1; SND1-sh2; n = 8).

**Additional file 3.** Original western blots from Figures 1 to 7. The original, uncropped western blot membranes or film scans are shown. Molecular weight markers are included when available. Figures 1E, 2B, 4B, 4C, 6B, 6F, 7C and Supplementary Fig. S2 included.

**Additional file 4.** Supplementary figure legends.

**Additional file 5.** Supplementary tables.

**Additional file 6.** Supplementary methods.

## Data Availability

All data generated or analysed during this study are included in this published article and its supplementary information files.

## References

[1] Sung H, Ferlay J, Siegel RL, Laversanne M, Soerjomataram I, Jemal A, Bray F (2021). Global cancer statistics 2020: GLOBOCAN estimates of incidence and mortality worldwide for 36 cancers in 185 Countries. CA.

[2] Siegel RL, Miller KD, Wagle NS, Jemal A (2023). Cancer statistics, 2023. CA.

[3] Weigelt B, Peterse JL (2005). van 't Veer LJ: Breast cancer metastasis: markers and models. Nat Rev Cancer.

[4] Hanna WM, Slodkowska E, Lu FI, Nafisi H, Nofech-Mozes S (2017). Comparative analysis of human epidermal growth factor receptor 2 testing in breast cancer according to 2007 and 2013 american society of clinical oncology/college of American pathologists guideline recommendations. J Clin Oncol.

[5] Wolff AC, Hammond ME, Hicks DG, Dowsett M, McShane LM, Allison KH, Allred DC, Bartlett JM, Bilous M, Fitzgibbons P (2013). Recommendations for human epidermal growth factor receptor 2 testing in breast cancer: American Society of Clinical Oncology/College of American Pathologists clinical practice guideline update. J Clin Oncol.

[6] Vihervuori H, Korpinen K, Autere TA, Repo H, Talvinen K, Kronqvist P (2022). Varying outcomes of triple-negative breast cancer in different age groups-prognostic value of clinical features and proliferation. Breast Cancer Res Treat.

[7] Li N, Wei J, Zhang Q, Liu B: Methyltransferase-like 3 enhances cell proliferation and cisplatin resistance in natural killer/T-cell lymphoma through promoting N6-methyladenosine modification and the stability of staphylococcal nuclease and Tudor domain-containing protein 1 mRNA. Anti-Cancer Drugs 2022. 10.1097/CAD.0000000000001433 36730541

[8] Zhao Y, Ren P, Yang Z, Wang L, Hu C: Inhibition of SND1 overcomes chemoresistance in bladder cancer cells by promoting ferroptosis. Oncol Rep 2023, 49(1). 10.3892/or.2022.8453 PMC9773013 36453257

[9] Ha C, Hu L, Ren Y, Yang J, Xin L (2022). SND1 confers chemoresistance to cisplatin-induced apoptosis by targeting GAS6-AKT in SKOV3 ovarian cancer cells. Med Oncol.

[10] Yoo BK, Santhekadur PK, Gredler R, Chen D, Emdad L, Bhutia S, Pannell L, Fisher PB, Sarkar D (2011). Increased RNA-induced silencing complex (RISC) activity contributes to hepatocellular carcinoma. Hepatology.

[11] Tsuchiya N, Nakagama H (2010). MicroRNA, SND1, and alterations in translational regulation in colon carcinogenesis. Mutat Res.

[12] Xin L, Zhao R, Lei J, Song J, Yu L, Gao R, Ha C, Ren Y, Liu X, Liu Y (2019). SND1 acts upstream of SLUG to regulate the epithelial-mesenchymal transition (EMT) in SKOV3 cells. FASEB J.

[13] Shen M, Wei Y, Kim H, Wan L, Jiang YZ, Hang X, Raba M, Remiszewski S, Rowicki M, Wu CG (2022). Small-molecule inhibitors that disrupt the MTDH-SND1 complex suppress breast cancer progression and metastasis. Nat Cancer.

[14] Shen M, Smith HA, Wei Y, Jiang YZ, Zhao S, Wang N, Rowicki M, Tang Y, Hang X, Wu S (2022). Pharmacological disruption of the MTDH-SND1 complex enhances tumor antigen presentation and synergizes with anti-PD-1 therapy in metastatic breast cancer. Nat Cancer.

[15] Yu L, Di Y, Xin L, Ren Y, Liu X, Sun X, Zhang W, Yao Z, Yang J (2017). SND1 acts as a novel gene transcription activator recognizing the conserved Motif domains of Smad promoters, inducing TGFbeta1 response and breast cancer metastasis. Oncogene.

[16] Yu L, Xu J, Liu J, Zhang H, Sun C, Wang Q, Shi C, Zhou X, Hua D, Luo W (2019). The novel chromatin architectural regulator SND1 promotes glioma proliferation and invasion and predicts the prognosis of patients. Neuro Oncol.

[17] Bucker L, Lehmann U: CDH1 (E-cadherin) Gene methylation in human breast cancer: critical appraisal of a long and twisted story. Cancers 2022, 14(18). 10.3390/cancers14184377 PMC9497067 36139537

[18] Ratze MAK, Koorman T, Sijnesael T, Bassey-Archibong B, van de Ven R, Enserink L, Visser D, Jaksani S, Viciano I, Bakker ERM (2022). Loss of E-cadherin leads to Id2-dependent inhibition of cell cycle progression in metastatic lobular breast cancer. Oncogene.

[19] O'Brien SJ, Fiechter C, Burton J, Hallion J, Paas M, Patel A, Rochet A, Scheurlen K, Gardner S, Eichenberger M (2021). Long non-coding RNA ZFAS1 is a major regulator of epithelial-mesenchymal transition through miR-200/ZEB1/E-cadherin, vimentin signaling in colon adenocarcinoma. Cell Death Discov.

[20] Li M, Rao X, Cui Y, Zhang L, Li X, Wang B, Zheng Y, Teng L, Zhou T, Zhuo W (2022). The keratin 17/YAP/IL6 axis contributes to E-cadherin loss and aggressiveness of diffuse gastric cancer. Oncogene.

[21] Kielbik M, Szulc-Kielbik I, Klink M: E-cadherin expression in relation to clinicopathological parameters and survival of patients with epithelial ovarian cancer. Int J Mol Sci 2022, 23(22): 14383 10.3390/ijms232214383 PMC9695266 36430858

[22] Luo M, Li J, Yang Q, Xu S, Zhang K, Chen J, Zhang S, Zheng S, Zhou J (2022). N4BP3 promotes breast cancer metastasis via NEDD4-mediated E-cadherin ubiquitination and degradation. Cancer Lett.

[23] Wijshake T, Zou Z, Chen B, Zhong L, Xiao G, Xie Y, Doench JG, Bennett L, Levine B: Tumor-suppressor function of Beclin 1 in breast cancer cells requires E-cadherin. In: Proceedings of the National Academy of Sciences of the United States of America 2021, 118(5). 10.1073/pnas.2020478118 PMC7865132 33495338

[24] Bai X, Jiang X, Liu Y, Wang Y, Song G, Qiu H, Zhang Q (2021). Kruppel-like factor 9 upregulates E-cadherin transcription and represses breast cancer invasion and metastasis. Am J Cancer Res.

[25] Karsten N, Kolben T, Mahner S, Beyer S, Meister S, Kuhn C, Schmoeckel E, Wuerstlein R, Harbeck N, Ditsch N (2022). The role of E-Cadherin expression in primary site of breast cancer. Arch Gynecol Obstet.

[26] Wang Y, Sun Y, Shang C, Chen L, Chen H, Wang D, Zeng X (2021). Distinct Ring1b complexes defined by DEAD-box helicases and EMT transcription factors synergistically enhance E-cadherin silencing in breast cancer. Cell Death Dis.

[27] Vareslija D, Ward E, Purcell SP, Cosgrove NS, Cocchiglia S, O'Halloran PJ, Charmsaz S, Bane FT, Brett FM, Farrell M (2021). Comparative analysis of the AIB1 interactome in breast cancer reveals MTA2 as a repressive partner which silences E-Cadherin to promote EMT and associates with a pro-metastatic phenotype. Oncogene.

[28] Lyko F (2018). The DNA methyltransferase family: a versatile toolkit for epigenetic regulation. Nat Rev Genet.

[29] Gao XN, Yan F, Lin J, Gao L, Lu XL, Wei SC, Shen N, Pang JX, Ning QY, Komeno Y (2015). AML1/ETO cooperates with HIF1alpha to promote leukemogenesis through DNMT3a transactivation. Leukemia.

[30] Kim G, Kim JY, Lim SC, Lee KY, Kim O, Choi HS (2018). SUV39H1/DNMT3A-dependent methylation of the RB1 promoter stimulates PIN1 expression and melanoma development. FASEB J.

[31] Stolzenburg S, Beltran AS, Swift-Scanlan T, Rivenbark AG, Rashwan R, Blancafort P (2015). Stable oncogenic silencing in vivo by programmable and targeted de novo DNA methylation in breast cancer. Oncogene.

[32] Li C, Xiong W, Liu X, Xiao W, Guo Y, Tan J, Li Y (2019). Hypomethylation at non-CpG/CpG sites in the promoter of HIF-1alpha gene combined with enhanced H3K9Ac modification contribute to maintain higher HIF-1alpha expression in breast cancer. Oncogenesis.

[33] Yu Z, Xiao Q, Zhao L, Ren J, Bai X, Sun M, Wu H, Liu X, Song Z, Yan Y (2015). DNA methyltransferase 1/3a overexpression in sporadic breast cancer is associated with reduced expression of estrogen receptor-alpha/breast cancer susceptibility gene 1 and poor prognosis. Mol Carcinog.

[34] Liu J, Pang Y, Wang H, Li Y, Sun X, Xu F, Ren H, Liu D (2016). miR-101 inhibits the proliferation and migration of breast cancer cells via downregulating the expression of DNA methyltransferase 3a. Chinese J Cell Mol Immunol.

[35] Iwamoto T, Niikura N, Ogiya R, Yasojima H, Watanabe KI, Kanbayashi C, Tsuneizumi M, Matsui A, Fujisawa T, Iwasa T (2019). Distinct gene expression profiles between primary breast cancers and brain metastases from pair-matched samples. Sci Rep.

[36] Diao C, Guo P, Yang W, Sun Y, Liao Y, Yan Y, Zhao A, Cai X, Hao J, Hu S (2021). SPT6 recruits SND1 to co-activate human telomerase reverse transcriptase to promote colon cancer progression. Mol Oncol.

[37] Jariwala N, Rajasekaran D, Mendoza RG, Shen XN, Siddiq A, Akiel MA, Robertson CL, Subler MA, Windle JJ, Fisher PB (2017). Oncogenic role of SND1 in development and progression of hepatocellular carcinoma. Can Res.

[38] Santhekadur PK, Das SK, Gredler R, Chen D, Srivastava J, Robertson C, Baldwin AS, Fisher PB, Sarkar D (2012). Multifunction protein staphylococcal nuclease domain containing 1 (SND1) promotes tumor angiogenesis in human hepatocellular carcinoma through novel pathway that involves nuclear factor kappaB and miR-221. J Biol Chem.

[39] Chen L, Song Y, Hou T, Li X, Cheng L, Li Y, Xing Y (2022). Circ_0004087 interaction with SND1 promotes docetaxel resistance in prostate cancer by boosting the mitosis error correction mechanism. J Exp Clin Cancer Res CR.

[40] Yu L, Liu X, Cui K, Di Y, Xin L, Sun X, Zhang W, Yang X, Wei M, Yao Z (2015). SND1 Acts downstream of TGFbeta1 and upstream of smurf1 to promote breast cancer metastasis. Can Res.

[41] Pasculli B, Barbano R, Parrella P (2018). Epigenetics of breast cancer: Biology and clinical implication in the era of precision medicine. Semin Cancer Biol.

[42] Yu J, Qin B, Moyer AM, Nowsheen S, Liu T, Qin S, Zhuang Y, Liu D, Lu SW, Kalari KR (2018). DNA methyltransferase expression in triple-negative breast cancer predicts sensitivity to decitabine. J Clin Investig.

[43] Teschendorff AE, Gao Y, Jones A, Ruebner M, Beckmann MW, Wachter DL, Fasching PA, Widschwendter M (2016). DNA methylation outliers in normal breast tissue identify field defects that are enriched in cancer. Nat Commun.

[44] Zhang J, Fan X, Zhou Y, Chen L, Rao H (2022). The PRMT5-LSD1 axis confers Slug dual transcriptional activities and promotes breast cancer progression. J Exp Clin Cancer Res CR.

[45] Chen F, Ding K, Priedigkeit N, Elangovan A, Levine KM, Carleton N, Savariau L, Atkinson JM, Oesterreich S, Lee AV (2021). Single-cell transcriptomic heterogeneity in invasive ductal and lobular breast cancer cells. Can Res.

[46] Aftimos P, Oliveira M, Irrthum A, Fumagalli D, Sotiriou C, Gal-Yam EN, Robson ME, Ndozeng J, Di Leo A, Ciruelos EM (2021). Genomic and transcriptomic analyses of breast cancer primaries and matched metastases in AURORA, the breast international group (BIG) molecular screening initiative. Cancer Discov.

