# Supplementary figures and images for: The chromatin architectural regulator SND1 mediates metastasis in triple-negative breast cancer by promoting CDH1 gene methylation
Source: Breast Cancer Res. 2023 Oct 26;25:129. doi: 10.1186/s13058-023-01731-3 (PMC10601136; doi:10.1186/s13058-023-01731-3)

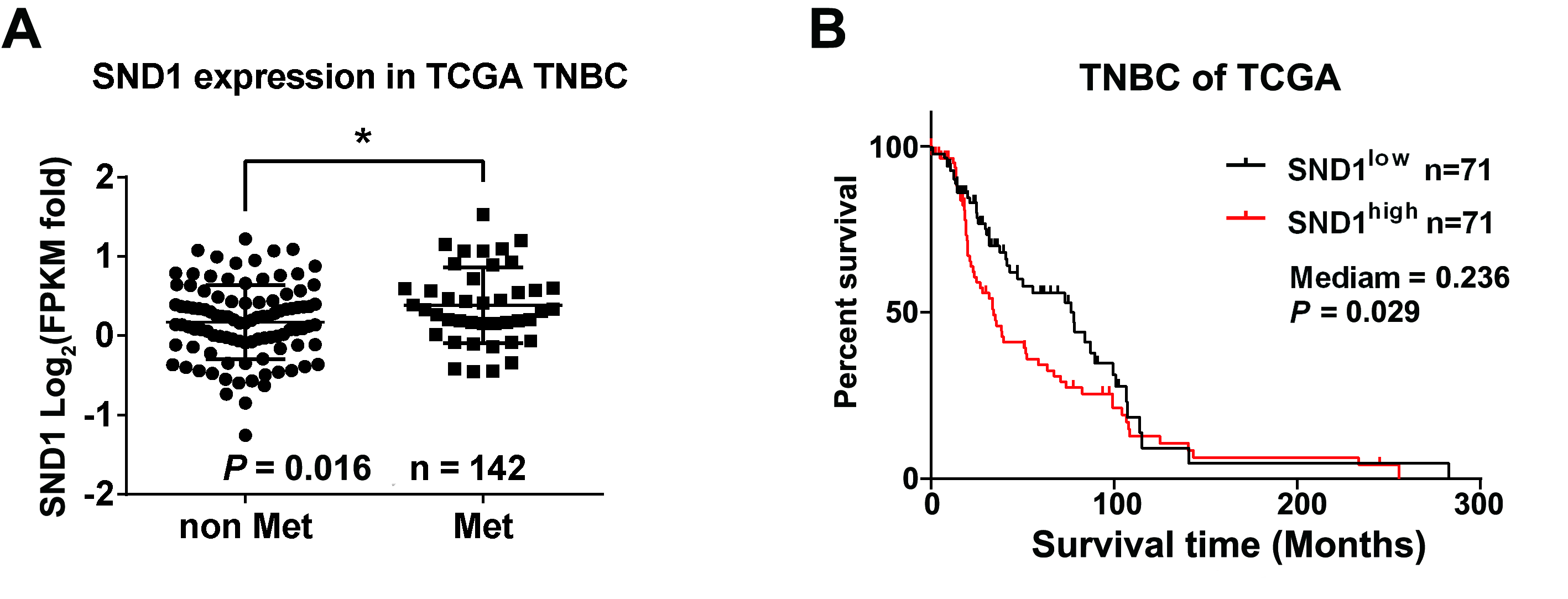

Supplement: Supplementary file 1 — Additional file 1. Supplementary Fig. S1. SND1 expression correlates to lymphatic metastasis and patient survival of TNBC. (A) Compare SND1 expression level between patients with (Met) or without (non Met) lymphatic metastasis from the TCGA TNBC (*, P = 0.016, n = 142). (B) K-M survival was plotted based on SND1 level in TNBC patients of TCGA. The low SND1 subgroup (n=71) showed more favourable prognosis than SND1 highly expression group (n = 71; P = 0.029). [file 13058_2023_1731_MOESM1_ESM.tif]

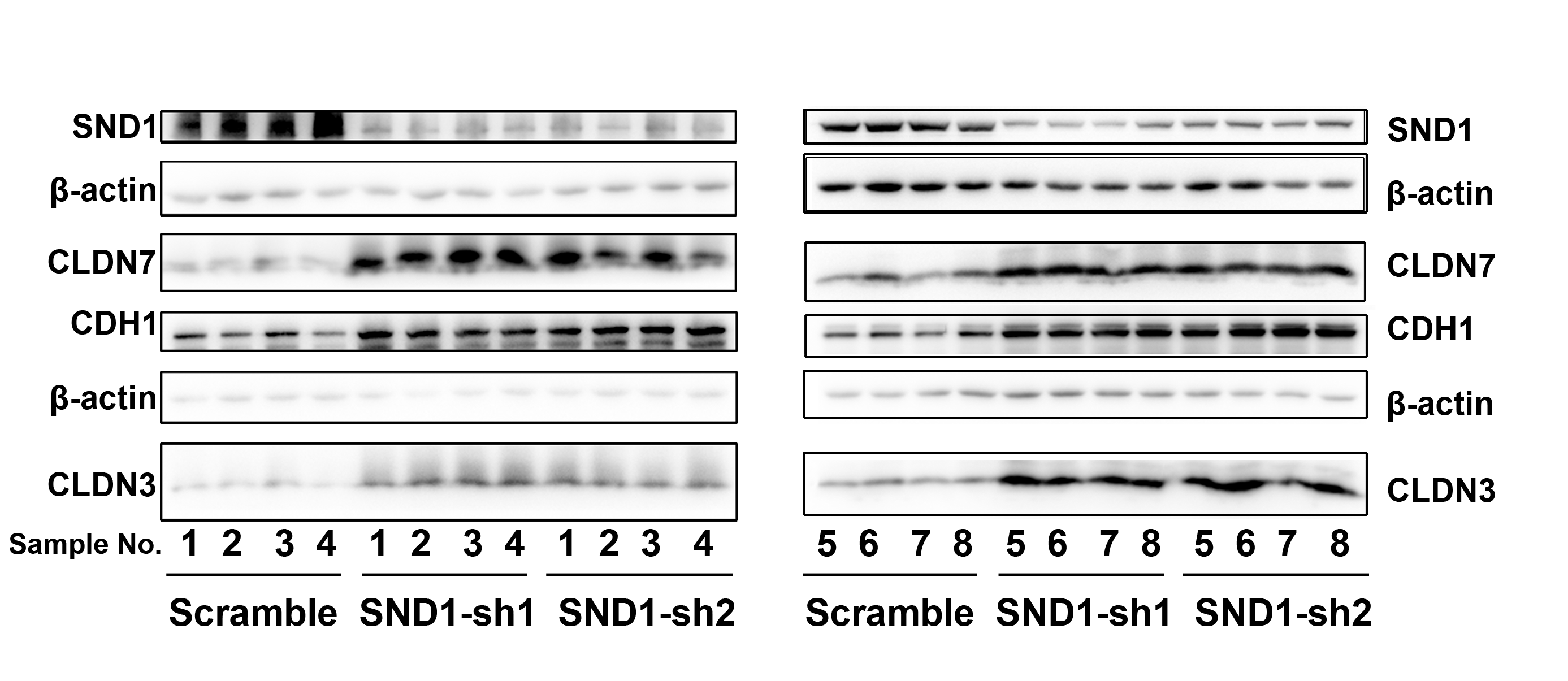

Supplement: Supplementary file 2 — Additional file 2. Supplementary Fig. S2. The metastasis tumour samples from mice were used to validate the expressions of SND1 and SND1 targets. Western results of SND1, CDH1, CLDN3 and CLDN7 expressions in metastasis tumour samples from mice transplanted with MDA-MB-231 cells of scramble control (scramble; n=8) or SND1 knocking down (SND1-sh1; SND1-sh2; n = 8). [file 13058_2023_1731_MOESM2_ESM.tif]
