# Supplementary material for: The chromatin architectural regulator SND1 mediates metastasis in triple-negative breast cancer by promoting CDH1 gene methylation
Source: Breast Cancer Res. 2023 Oct 26;25:129. doi: 10.1186/s13058-023-01731-3 (PMC10601136; doi:10.1186/s13058-023-01731-3)
Supplement: Supplementary file 4 — Additional file 4. Supplementary figure legends. [file 13058_2023_1731_MOESM4_ESM.docx]

**Supplementary Figure Legends**

**Supplementary Figure S1 SND1 expression correlates to lymphatic metastasis and patient survival of TNBC**. **(A)** Compare SND1 expression level between patients with (Met) or without (non Met) lymphatic metastasis from the TCGA TNBC (*, *P* = 0.016, n = 142). **(B)** K-M survival was plotted based on SND1 level in TNBC patients of TCGA. The low SND1 subgroup (n=71) showed more favourable prognosis than SND1 highly expression group (n = 71; *P* = 0.029).

**Supplementary Figure S2 The metastasis tumour samples from mice were used to validate the expressions of SND1 and SND1 targets.** Western results of SND1, CDH1, CLDN3 and CLDN7 expressions in metastasis tumour samples from mice transplanted with MDA-MB-231 cells of scramble control (scramble; n=8) or SND1 knocking down (SND1-sh1; SND1-sh2; n=8).
