# Supplementary material for: The chromatin architectural regulator SND1 mediates metastasis in triple-negative breast cancer by promoting CDH1 gene methylation
Source: Breast Cancer Res. 2023 Oct 26;25:129. doi: 10.1186/s13058-023-01731-3 (PMC10601136; doi:10.1186/s13058-023-01731-3)
Supplement: Supplementary file 5 — Additional file 5. Supplementary tables. [file 13058_2023_1731_MOESM5_ESM.docx]

**Supplementary Table 1.** The clinical features of patients with TNBC in this study

| **Feature** | **The source of patients with TNBC** | |
| --- | --- | --- |
|  | **TMUGH (n=58)** | **TCGA (n=142)** |
| Age (Year, Mean ± SD) | 55.2±9.4 | 56.4±12.6 |
| TMN stage |  |  |
| Stage I | 8 | 24 |
| Stage II | 14 | 73 |
| Stage III | 29 | 41 |
| Stage IV | 7 | 4 |

Abbreviation: SD, standard deviation.

**Supplementary Table 2.** Multivariate analysis for DFS and OS in patients with TNBC from TMUGH

| **Factors** | DFS | |  | OS | |
| --- | --- | --- | --- | --- | --- |
|  | HR(95%CI) | *P* |  | HR(95%CI) | *P* |
| **Age** | 1.133(0.902-1.235) | 0.061 |  | 1.224(1.109-1.317) | 0.031 |
| **TMN stage** | 1.327(1.224-1.392) | <0.001 |  | 1.182(1.159-1.662) | <0.001 |
| **MKI67 LI** | 1.112(1.092-1.201) | 0.019 |  | 1.204(1.289-1.343) | 0.027 |
| **SND1 protein LI** | 1.188(1.117-1.262) | <0.001 |  | 1.201(1.188-1.273) | <0.001 |

Abbreviations: HR, hazard ratio; CI, confidence interval; LI, labeling index.

**Supplementary Table 3.** Multivariate analysis for DFS and OS in patients with TNBC from TCGA

| **Factors** | DFS | |  | OS | |
| --- | --- | --- | --- | --- | --- |
|  | HR(95%CI) | *P* |  | HR(95%CI) | *P* |
| **Age** | 1.159(0.286-4.695) | 0.836 |  | 1.127(0.294-3.916) | 0.715 |
| **TMN stage** | 2.427(1.901-2.903) | <0.001 |  | 2.214(1.708-4.537) | <0.001 |
| **MKI67 LI** | 2.890(1.488-5.618) | 0.002 |  | 2.801(1.328-5.917) | 0.007 |
| **SND1 mRNA** **log_2_ (fpkm fold)** | 2.573(1.662-3.983) | <0.001 |  | 1.852(1.267-2.861) | <0.001 |

Abbreviations: HR, hazard ratio; CI, confidence interval; LI, labeling index.

**Supplementary Table 4.** Primers and probes used for this study

| **qRT-PCR primers** | |  | **Sequence** |
| --- | --- | --- | --- |
| **SND1** | | **forward** | **5’-CTTATCACCTTCTTGCTTGCAG-3’** |
|  |  | **reverse** | **5’-AAAGTGTAGCTTCCTCGCTGA-3’** |
| **DNMT3A** | | **forward** | **5’-CTGACAGAGGCACCGTTCAC-3’** |
|  |  | **reverse** | **5’-TATCGTGGTCTTTGGAGGCG-3’** |
| **GAPDH** | | **forward** | **5’-TGCACCACCAACTGCTTAGC-3’** |
|  |  | **reverse** | **5’-GGCATGGACTGTGGTCATGAG-3’** |
| **ACTB** | | **forward** | **5’-GTCATTCCAAATATGAGATGCGT-3’** |
|  |  | **reverse** | **5’-GCTATCACCTCCCCTGTGTG-3’** |
| **CDH1** | | **forward** | **5’-TGGTACCTGGCAAGATGCAG-3’** |
|  |  | **reverse** | **5’-GGGGGCTTCATTCACATCCA-3’** |
| **ChIP primers** | |  | **Sequence** |
| **DNMT3A-promoter-1** | | **forward** | **5’- CTCGGCTCACTAAAATGTTCGC-3’** |
|  |  | **reverse** | **5’- TAAAAGGTCCGGGTGTGATGG-3’** |
| **DNMT3A-promoter-2** | | **forward** | **5’- ACCCACCATTCCACTTCTGTC-3’** |
|  |  | **reverse** | **5’- CAAAAGTGGAGGCAACCTGATG-3’** |
| **DNMT3A-promoter-3** | | **forward** | **5’- TTGACCCCAGCAACCCATAC-3’** |
|  |  | **reverse** | **5’- CTCAGGGCCATGATGAGCAA-3’** |
| **DNMT3A-promoter-4** | | **forward** | **5’- CACCTCAAGGTACGGGGAAC-3’** |
|  |  | **reverse** | **5’- AGAGGCCACTCTGCTCTACA-3’** |
| **DNMT3A-promoter-5** | | **forward** | **5’- CAGAAGCAGCGCCAGGTAG-3’** |
|  |  | **reverse** | **5’- CTCGAGTCGTAGCCGAGC-3’** |
| **CDH1-promoter-1** | | **forward** | **5’- AACATGGTGAAACCCCGTCTG-3’** |
|  |  | **reverse** | **5’- TCTCGGCTCACTGCAACCTCC-3’** |
| **CDH1-promoter-2** | | **forward** | **5’- GTGGAATCAGAACCGTGCAG-3’** |
|  |  | **reverse** | **5’- CATAGACGCGGTGACCCTC-3’** |
| **CDH1-promoter-3** | | **forward** | **5’- GAACCCTCAGCCAATCAGCGGTA-3’** |
|  |  | **reverse** | **5’- ACTGACTTCCGCAAGCTCACA-3’** |
| **CDH1-promoter-4** | | **forward** | **5’- GATCCCCTGACTTGCGAGG-3’** |
|  |  | **reverse** | **5’- CCTCAGGACCCGAACTTTCTT-3’** |
| **CDH1-promoter-5** | | **forward** | **5’- GGTGCATTCCCGGTCTAAGG-3’** |
|  |  | **reverse** | **5’- TCAAGACCTAGCCCACCGTT-3’** |
| **EMSA probes** | | | **Sequence** |
| **DNMT3A-EMSA-probe full length** | | | **5’- TGATCTCGGCTCACTTGATCTCGGCTCACT-3’** |
| **DNMT3A-EMSA-probe-mut 5’** | | | **5’- TAATATAGGCTCACTTAATATAGGCTCACT -3’** |
| **DNMT3A-EMSA-probe-mut 3’** | | | **5’- TGATCTCAAATAAATTGATCTCAAATAAAT-3’** |
| **DNMT3A-EMSA-probe-mut 5+3’** | | | **5’- TAATATAAAATAAATTAATATAAAATAAAT-3’** |
| **3C Primers** | | | **Sequence** |
| **3C-1** |  | | **5’- GCTCACTAAAATGTTCGCCTCC-3’** |
| **3C-2** |  | | **5’- TCAGGTTGCCTCCACTTTTG-3’** |
| **3C-3** |  | | **5’- TTCAAAAGTGTGGTGTGGGC-3’** |
| **3C-4** |  | | **5’- TGAATCCTGACTCTGCCACTTC-3’** |
| **3C-5** |  | | **5’- TGGTGGAATTAGACAGAGGTGC-3’** |
| **3C-6** |  | | **5’-CTCTGAGTGGCCGGGAGCGCG-3’** |

**Supplementary Table 5.** Reagents or resource used for this study .

| **Reagent or resource** | **Source** | **Identifier** |
| --- | --- | --- |
| **Antibodies** |  |  |
| Anti-SND1 antibody | Santa Cruz | sc-166676 |
| Anti-CDH1 antibody | CST | #3195 |
| Anti-CDH1 antibody | Thermo Fisher | 13-1700 |
| Anti-DNMT3A antibody | CST | #32578 |
| Anti-β-actin antibody | Abcam | ab8226 |
| Anti-Histone H3 (acetyl K9) antibody | Abcam | ab32129 |
| Anti-Histone H3 (acetyl K27) antibody | Abcam | ab4729 |
| Anti-5-methylcytosine antibody | Abcam | ab10805 |
| Normal rabbit IgG | Proteintech | 30000-0-AP |
| Normal mouse IgG | Proteintech | B900620 |
| HRP-conjugated Donkey Anti-Rabbit IgG(H+L) | Proteintech | SA00001-9 |
| HRP-conjugated Donkey Anti-Mouse IgG(H+L) | Proteintech | SA00001-8 |
| Biotinylated secondary antibody | Beyotime | A0286 |
| HRP-labeled streptavidin | Beyotime | A0303 |
| Alexa Fluor^TM^ 488 donkey anti-rabbit IgG | Thermo Fisher | R37118 |
| Alexa Fluor^TM^ 546 donkey anti-mouse IgG | Thermo Fisher | A10036 |
| Protein A/G magnetic beads | Millipore | LSKMAGAG02 |
| **Cell and Cell culture related reagents** |  | |
| HEK293T | ATCC | CRL-3216 |
| MDA-MB-231 | ATCC | HTB-26 |
| BT549 | ATCC | HTB-122 |
| Dulbecco’s Modified Eagle’s Medium | Biological Industries | 01-052-1ACS |
| Leibovitz L-15 medium | Hyclone | SH30525.01 |
| RPMI-1640 | Biological Industries | 01-100-1A |
| Foetal bovine serum | Biological Industries | 04-010-1A |
| Insulin | Beyotime | P3376 |
| Transfection Reagent TransIT-Lenti | Mirus | MIR 6610 |
| Polybrene | Beyotime | C0351 |
| Puromycin | Solabio | P8230 |
| Lipofectamine 3000 | Thermo Fisher | L3000015 |
| Matrigel | Corning, | 356231 |
| DAPI | Beyotime | C1002 |

| **Critical commercial assays** |  |  |
| --- | --- | --- |
| RIPA buffer | Solabio | R0010 |
| Protease inhibitor cocktail | Roche | 04693132001 |
| TRIzol LS Reagent | Invitrogen | 10296010 |
| RevertAid First Strand cDNA Synthesis Kit | Thermo Fisher | K1622 |
| FastStart Universal SYBR Green Master | Sigma-Aldrich | 4913850001 |
| Transwell inserts | Millipore, | PTEP24H48 |
| EZ-Magna-ChIP™-Kit | Sigma-Aldrich | 17-10086 |
| Dual-Luciferase Reporter Assay Kit | Promega | E1910 |
| DNA extraction kit | Solarbio | D1700-100T |
| EpiTectPlus DNA Bisulfite Kit | QIAGEN | 59124 |
| MinElute Reaction Cleanup Kit | QIAGEN | 28206 |
| PCR Purification Miniprep kit | Biomiga | DC3511 |
| DAB stain kit | Beyotime | P0203 |
| DpnII | NEB | R0543S |
| T4 DNA ligase | NEB | M0202S |
| D-luciferin | MEM | HY-12591A |
| LightShift Chemiluminescent EMSA kit | Thermo Fisher | 20148 |
| **Vectors** |  |  |
| pLKO.1 | Sigma-Aldrich | SHC001 |
| pLV-IRES | Clontech | 632183 |
| pGL3 | Promega | E1751 |
| **Software** |  |  |
| SPSS | IBM | version 21.0 |

**Supplementary Table 6.** Data of cDNA microarray

| **Gene name** | **control** | **SND1-sh1** | **SND1-sh2** |
| --- | --- | --- | --- |
| **CDH1** | **0.120706** | **1.298315** | **1.580979** |
| **HTRA1** | **2.420017** | **0.209893** | **0.37009** |
| **ADRBK1** | **2.38326** | **0.339207** | **0.277533** |
| **SND1** | **2.502147** | **0.248396** | **0.249457** |
| **ST14** | **0.312505** | **1.257816** | **1.42968** |
| **SMAD3** | **1.719086** | **0.61681** | **0.664103** |
| **DNMT3A** | **1.802127** | **0.48056** | **0.717313** |
| **SERPINA3** | **2.738276** | **0.058366** | **0.203359** |
| **CLDN7** | **0.143226** | **1.372998** | **1.483776** |
| **SLC16A3** | **0.376759** | **1.397796** | **1.225445** |
| **IFIT1** | **2.0112** | **0.480693** | **0.508107** |
| **GALNT3** | **0.241442** | **1.383506** | **1.375052** |
| **CDH2** | **1.977262** | **0.516172** | **0.506566** |
| **ALDH5A1** | **1.672389** | **0.630579** | **0.697032** |
| **TMOD1** | **1.801793** | **0.509111** | **0.689097** |
| **CLDN3** | **0.369255** | **1.235174** | **1.395571** |
| **CCL4** | **2.247945** | **0.40274** | **0.349315** |
| **SMAD2** | **1.622353** | **0.684321** | **0.693326** |
| **MUC4** | **0.36028** | **1.473953** | **1.165767** |
| **IGFBP3** | **0.479417** | **1.342202** | **1.17838** |
| **GJB3** | **0.405427** | **1.332004** | **1.262569** |
| **OAS1** | **1.72176** | **0.665836** | **0.612404** |
| **GAL3ST1** | **1.605038** | **0.636267** | **0.758695** |
| **ARVCF** | **2.174777** | **0.676266** | **0.148957** |
| **DNMT3B** | **2.2705** | **0.511725** | **0.217774** |
| **SLC6A6** | **0.509311** | **1.212478** | **1.278211** |
| **PPIL2** | **2.154185** | **0.239773** | **0.606042** |
| **BNC1** | **0.286021** | **1.224548** | **1.489431** |
| **OVOL1** | **2.26952** | **0.556969** | **0.173511** |
| **WISP1** | **2.136407** | **0.096061** | **0.767531** |
| **CELA2B** | **0.251004** | **1.468876** | **1.28012** |
| **OR7C2** | **1.74383** | **0.630097** | **0.626073** |
| **SYK** | **0.0934** | **1.109589** | **1.797011** |
| **NR2F1** | **1.916834** | **0.420772** | **0.662395** |
| **ADARB2** | **0.210596** | **1.382781** | **1.406623** |
| **OLR1** | **0.281415** | **1.507348** | **1.211237** |
| **MAPK13** | **0.556787** | **1.219936** | **1.223276** |
| **CRYAA** | **1.961977** | **0.242395** | **0.795627** |
| **ITSN2** | **0.073931** | **1.244504** | **1.681566** |
| **PTGER3** | **0.187723** | **1.4559** | **1.356377** |
| **MGLL** | **0.209938** | **1.65101** | **1.139052** |
| **CDC25A** | **0.338279** | **1.301187** | **1.360534** |
| **PAQR5** | **0.464873** | **1.239849** | **1.295278** |
| **NEFL** | **1.669163** | **0.725123** | **0.605714** |
| **NPR1** | **0.361353** | **1.545157** | **1.09349** |
| **BSPRY** | **0.263033** | **1.498922** | **1.238045** |
| **DAPP1** | **0.291525** | **1.555791** | **1.152683** |
| **CXCL16** | **0.435668** | **1.273469** | **1.290863** |
