# Supplementary material for: The chromatin architectural regulator SND1 mediates metastasis in triple-negative breast cancer by promoting CDH1 gene methylation
Source: Breast Cancer Res. 2023 Oct 26;25:129. doi: 10.1186/s13058-023-01731-3 (PMC10601136; doi:10.1186/s13058-023-01731-3)
Supplement: Supplementary file 6 — Additional file 6. Supplementary methods. [file 13058_2023_1731_MOESM6_ESM.docx]

**Supplementary Methods**

**Cell culture and phenotype assay**

The HEK293T (ATCC CRL-3216), MDA-MB-231 (ATCC HTB-26) and BT549 (ATCC HTB-122) cell lines were purchased from the American Type Culture Collection. The HEK293T was cultured in Dulbecco’s Modified Eagle’s Medium (Biological Industries 01-052-1ACS) with 10% foetal bovine serum (FBS, Biological Industries 04-010-1A) at a temperature of 37 °C without CO_2_. The MDA-MB-231 was cultured in Leibovitz L-15 medium (Hyclone SH30525.01) supplemented with 10% FBS (Biological Industries 04-010-1A) at a temperature of 37 °C without CO_2_. The BT549 was cultured in RPMI-1640 (Biological Industries 01-100-1A) containing 10% FBS (Biological Industries 04-010-1A) and insulin (Beyotime P3376, 0.023 IU/mL) at 37 °C with 5% CO_2_.

The membranes in Transwell inserts (Millipore, PTEP24H48) were precoated with Matrigel (Corning, 356231), and 5x10^5^ MDA-MB-231 and BT-549 cells were resuspended with 200 µL medium without FBS and loaded to insert cells of the Boyden chambers. After incubation for 24 hours, infiltrated cancer cells were treated by 4% paraformaldehyde (Sigma-Aldrich V900894) for fixation; then the cells were further stained by 0.2% crystal violet (MCE HY-B0324A) for cell counting.

Three vertical scratch wounds were made in each well of 6-well plates containing two breast cancer cells at 95% confluence. The images of the wounds were captured via microscopy at 0 h and 24 h, the migration distance was measured and relative migration (%) was calculated.

**Plasmids, lentiviruses, and construction of cell lines with stable expression**

The lentiviral vector pLKO.1 was obtained from Sigma-Aldrich (SHC001). Two SND1 shRNA plasmids were constructed as pLKO-SND1-sh1 (5’-TGTGGCTCCCACAGCTAATTT-3’) and pLKO-SND1-sh2 (5’-TCTCGTCTCAAACTCTATTTG-3’). The full length human SND1 and DNMT3A was cloned into pLV-IRES expression vector (Clontech 632183) and named as pLV-IRES-SND1 and pLV-IRES-DNMT3A. The full length and mutated promoter sequences of DNMT3A were cloned into pGL3 reporter vector (Promega E1751) and named as pGL3-DNMT3A full length, pGL3-DNMT3A ΔTSS, pGL3-DNMT3A ΔR1, pGL3-DNMT3A R2 and pGL3-DNMT3A ΔR1+R2. Lentiviruses were produced in HEK293T cells with Transfection Reagent TransIT-Lenti (Mirus MIR 6610). After 48 hours, the virus media was filtered and store at -80℃. For infection, the viruses were thawed and incubated to cell lines with 10ng/ml polybrene (Beyotime C0351).

Both BT549 and MDA-MB-231 cells were infected by the aforementioned shRNA and overexpression lentivirus, and 1 µg/mL puromycin (Solabio P8230) was used to select the stably transfected subclones (scramble, SND1-sh1, SND1-sh2, SND1-sh1+SND1 res, SND1-sh2+SND1 res, SND1-sh1+DNMT3A, SND1-sh2+DNMT3A).

**DNA electrophoretic mobility shift assay (EMSA)**

The Supplementary Table 3 lists all the *DNMT3A* promoter probes used in this study (biotin-labelled, unlabelled and mutated). A LightShift Chemiluminescent EMSA kit (Thermo 20148) was used for EMSA, and the reaction was conducted according to the manufacturer’s instructions. The reaction mixtures included the following: a negative control mixture (containing 20 fmol of labelling probe), the binding reaction mixture (containing 10 μg of SND1 protein and 20 fmol of labelling probe), the 5’ mutated probe reaction mixture (containing 10 μg of SND1 protein, 20 fmol of 5’ mutated probe), the 3’ mutated probe reaction mixture (containing 10 μg of SND1 protein, 20 fmol of 3’ mutated probe), the 5’+3’ mutated probe reaction mixture (containing 10 μg of SND1 protein, 20 fmol of 5’+3’ mutated probe), the unlabelled probe competition reaction mixture (containing 10 μg of SND1 protein, 20 fmol of labelling probe and 1 nmol of label-free probe). After 30 minutes of incubation, the samples were subjected to 7% native PAGE (85 V, 45 minutes).

**cDNA microarray and data analysis**

Log-phase MDA-MB-231 cells were collected, and TRIzol reagent (Invitrogen 10296010) was used to extract total RNA from cells. cDNA was generated using a RevertAid cDNA Synthesis Kit (Thermo, K1622). cDNA was hybridized to Affymetrix HG-U133 Plus 2.0 arrays (Affymetrix, Thermo, Rockford, USA) according to standard protocol (n=3 for each group). Gene expression data were analysed by the Bioconductor package of R software, and one-way ANOVA was used for multiple group comparison. The false discovery rate (FDR) of multiple testing corrections was performed by the Benjamini and Hochberg method. Significant expression changes were filtered at >2-fold and a FDR <0.05 between experiments. These genes were then subjected to an unsupervised 2-way average linkage hierarchical cluster analysis with uncentered correlation as the similarity metric using Cluster 3.0 software; visualizing heat maps were generated by Java Tree view software. The functional annotation was done using the Gene Ontology database and DAVID software on differentially regulated genes.

**qRT-PCR and western blot**

TRIzol LS Reagent (Invitrogen 10296010) was used for total RNA purification of breast cancer cells, the RevertAid First Strand cDNA Synthesis Kit (Thermo Fisher K1622) was used for reverse transcription and cDNA synthesis. The information of all primers was described in Table 3 (GENEWIZ). The FastStart Universal SYBR Green Master (Sigma-Aldrich 4913850001) and StepOne Real-Time PCR System (Thermo Fisher) were used to perform qPCR basing on the ΔΔCT method. The β-actin was set as internal reference gent to quantify mRNA relative levels. All primers were listed at Supplementary Table 3

Protein samples dissolved in RIPA buffer (Solabio R0010) with Roche protease inhibitor cocktail (Roche 04693132001) were loaded to 8% SDS‒PAGE for electrophoresis, and analysed by immunoblotting with the following antibodies: CDH1 (CST # 3195, dilution ratio 1:1000), DNMT3A (CST, # 32578 dilution ratio 1:1000), SND1 (Santa Cruz sc-166676 dilution ratio 1:750) and β-actin (Abcam, ab8226 dilution ratio 1:2000).

**Chromatin immunoprecipitation (ChIP) and chromosome conformation capture (3C) assay**

The chromatin was precipitated via mouse IgG (Proteintech B900620, 10 µg antibody per 500µl lysate) anti-Histone H3 (acetyl K9) antibody (Abcam ab32129), anti-Histone H3 (acetyl K27) antibody (Abcam ab4729) and SND1 antibody (Santa Cruz sc-166676X, 10 µg antibody per 500µl lysate) respectively. Then EZ-Magna-ChIP™-Kit (Sigma-Aldrich, 17-10086) was used to purify chromatin for further detection as the manual instruction. Finally, the relative enrichment of purified immunoprecipitated DNA samples was determined by qPCR. The sequences of the primers specific for the DNMT3A promoter are listed in Supplementary Table 3.

After formaldehyde cross-linking, nuclei were treated with DpnII (NEB R0543S), and fragments were ligated via T4 DNA ligase (NEB M0202S). The cross-linking efficiency between anchor sites and other sites was detected by qPCR. The PCR product resulting from amplification of the DNMT3A promoter sequence including transcription start site was also digested with DpnII, and the digestion product was relinked by incubation with an excess of DNA ligase and used as a standard. The rates of cross-linking between different sites were normalized to that of the standard. All primers were listed at Supplementary Table 3.

**Immunofluorescence (IF)**

BT549 and MDA-MB-231 were fixed with 4% paraformaldehyde (Sigma-Aldrich V900894), then the cells were further treated with 0.2% Triton X-100 (Sigma-Aldrich X100PC) cytomembrane permeabilization and 5% BSA (Sangon Biotech A500023) blocking. CDH1 was detected using a mouse monoclonal antibody (Thermo Fisher 13-1700 dilution ratio 1:75), DNMT3A was detected using a rabbit monoclonal antibody (CST, # 32578 dilution ratio 1:50), the fluorescence secondary antibodies were Alexa Fluor^TM^ 488 donkey anti-rabbit IgG (Thermo Fisher R37118 dilution ratio 1:100) and Alexa Fluor^TM^ 546 donkey anti-mouse IgG (Thermo Fisher A10036 dilution ratio 1:100). The nuclei were stained by DAPI (Beyotime C1002). All the immunofluorescence images were acquired by confocal microscope (FV1200; Olympus, Tokyo, Japan). The mean of positive cell percentage was calculated by five random selected 400X fields.

**Bisulfite sequencing PCR**

The genomic DNA of MDA-MB-231 was extracted with DNA extraction kit (Solarbio D1700-100T) to detect CpG methylation on the CDH1 gene promoter. Then, the purified DNA was processed through EpiTectPlus DNA Bisulfite Kit (QIAGEN, 59124) by the protocol, and the products were purified and collected by using a MinElute Reaction Cleanup Kit (QIAGEN 28206). The purified products were amplified by PCR and purified with the PCR Purification Miniprep kit (Biomiga, DC3511). Then the PCR product fragments were sub-cloned into sequencing vector and preformed Sanger sequencing (GENEWIZ). The sequencing data were analysed with the BiQ Analyzer tool.
